# Activation of NMDA receptors in brain endothelial cells increases transcellular permeability

**DOI:** 10.1186/s12987-022-00364-6

**Published:** 2022-09-06

**Authors:** Kyu-Sung Kim, Min Tae Jeon, Eun Seon Kim, Chan Hee Lee, Do-Geun Kim

**Affiliations:** 1 Neuroimmunology Lab, Dementia Research Group, Korea Brain Research Institute, Daegu, 41062 South Korea; 2 Department of Brain Science, Daegu Gyeongbuk Institute of Science & Technology (DGIST), Daegu, South Korea

## Abstract

Neurovascular coupling is a precise mechanism that induces increased blood flow to activated brain regions, thereby providing oxygen and glucose. In this study, we hypothesized that N-methyl-D-aspartate (NMDA) receptor signaling, the most well characterized neurotransmitter signaling system which regulates delivery of essential molecules through the blood–brain barrier (BBB). Upon application of NMDA in both in vitro and in vivo models, increased delivery of bioactive molecules that was mediated through modulation of molecules involved in molecular delivery, including clathrin and caveolin were observed. Also, NMDA activation induced structural changes in the BBB and increased transcellular permeability that showed regional heterogeneity in its responses. Moreover, NMDA receptor activation increased endosomal trafficking and facilitated inactivation of lysosomal pathways and consequently increased molecular delivery mediated by activation of calmodulin-dependent protein kinase II (CaMKII) and RhoA/protein kinase C (PKC). Subsequent in vivo experiments using mice specifically lacking NMDA receptor subunit 1 in endothelial cells showed decreased neuronal density in the brain cortex, suggesting that a deficiency in NMDA receptor signaling in brain endothelial cells induces neuronal losses. Together, these results highlight the importance of NMDA-receptor-mediated signaling in the regulation of BBB permeability that surprisingly also affected CD31 staining.

**Supplementary Information:**

The online version contains supplementary material available at 10.1186/s12987-022-00364-6.

## Introduction

Brain health is critically dependent on rapid delivery of nutrients, blood gas exchange, and waste removal [1, 2]. The brain has specialized vascular structures that are distinct from those in the peripheral vasculature, as reviewed by Daneman and Prat [3], including tight and adherens junctions between capillary endothelial cells that increase their mechanical and transcellular electrical resistance [4]. Brain endothelial cells are also covered with matrix proteins, pericytes, and astrocytic end-foot processes that further increase their resistance [5]. The blood–brain barrier (BBB) is a specialized vascular structure that protects brain parenchyma against peripheral insults. The BBB tightly regulates entry of molecules into and out of the CNS [2]. It is still elusive how the brain controls the entry of many different types of molecules. Defining the molecular pathways that affect the permeability of the BBB to therapeutic agents is important for treating brain cancer and neurodegenerative diseases [6, 7].

Neurovascular coupling is the process by which active neurons signal and coordinate fast changes in the cytoarchitecture, affecting the permeability of the microvasculature to increase regional blood flow. These phenomena have been documented by positron emission tomography studies using [18F]-fluoro-2-deoxy-d-glucose and by blood oxygen measurements using functional magnetic resonance imaging [8, 9]; thus, providing evidence of a sophisticated molecular delivery system that controls which molecules can reach the brain [10, 11]. How this physiological phenomenon occurs is still unclear despite the experimental evidence that demonstrates its existence [11, 12]. Traditionally, neurovascular coupling has been suggested to occur via vasodilation mediated by nitric oxide or prostaglandin released from astrocytes that are activated by neurotransmitters, rather than by direct activation of brain endothelial cells by neurotransmitters [11, 12]. However, recent findings from several groups have revealed the existence of neurotransmitter receptors on brain endothelial cells [4, 13]. Stimulation of neurotransmitter receptors on the brain side of the endothelial cells appears to mediate their vasodilation and nitric oxide (NO) production, suggesting that activated neuronal release of neurotransmitters and other signaling molecules may indeed mediate the vasodilatory effect [10, 13]. However, the concept of neurovascular coupling mainly focuses on changes in arteriolar blood flow, rather than on changes in the molecular delivery at the capillary level, where substances actually cross the physical and biological barriers. Our lack of understanding of this process warrants research into the mechanisms by which neurotransmitters induce changes in molecular delivery at the capillary level.

Molecular delivery to the brain is mediated through both paracellular and transcellular pathways, as reviewed by Villasenor et al. [14]. The paracellular pathway utilizes disruption of cell-to-cell junctions, whereas the transcellular pathway is mediated by transporters or transcytosis [14]. Paracellular resistance is regulated by changes in tight and adherens junctions mediated by actin-cytoskeletal reorganization, which is governed by small GTPases, including RhoA [15, 16]. It has been reported that transcytosis is mediated by clathrin-mediated endocytosis, receptor-mediated transcytosis, and caveolin-mediated transcytosis [17–20]. Molecules that enter brain endothelial cells through transcytosis do so by entry via the endosomal trafficking pathway. Recent studies have shown that the fate of macromolecules passing brain endothelial cells is determined by whether they can escape the late endosomal trafficking pathway. Molecules that enter endosomes fuse with lysosomes and are degraded [21], while molecules that persist in early endosomes can be delivered to the brain when they fuse with the cell membrane and are delivered to the brain via exocytosis [14]. The mechanisms governing transcytosis and its regulation remain largely unknown.

The roles of N-methyl-D-aspartate (NMDA) receptor activation are well studied, especially with respect to synaptic plasticity through long-term potentiation (LTP) and long-term depression (LTD) [22, 23]. Multimeric NMDA receptor complexes are composed of three different types of subunits called GluN1-3. GluN1, GluN2, and GluN3 have 8, 4, and 2 different types of splicing variants, respectively [24, 25]. Activation of NMDA receptors is mediated by binding of glutamate to GluN2 subunits and binding of glycine to GluN1 and GluN3 subunits [22, 26, 27]. Depolarization is achieved by activation of the AMPA receptor, which induces removal of Mg^2+^ from the cationic channel and subsequently induces an influx of calcium ions. This calcium ion influx mediates various physiological changes, including coordination of synaptic plasticity in the brain, as a second messenger [26, 28]. In neuronal cells, NMDA receptor activation induces activity of calmodulin-dependent protein kinase II (CaMKII). This in turn mediates the activation of RhoA and protein kinase C (PKC), increasing the recirculation of endosomal receptors and thereby enhancing LTP [29, 30]. Traditionally, it has been thought that NMDA receptors are only expressed in neuronal cells, and their role was thought to be confined to neuronal stimulation. However, in vitro and in vivo studies have also observed expression of GluN subunits in glial cells and other non-neuronal cells [31–33], including brain endothelial cells [34, 35]. Brain endothelial cell NMDA receptors, which are activated by glutamate and glycine, are thought to participate in the opening of the BBB [36, 37]. The concept of neurovascular coupling proposes that neural activity triggers increased blood flow and hyperemia to stimulated brain regions [38, 39].Considering the low permeability of the BBB and the requirement for high-velocity delivery of molecules to stimulated brain regions, it is logical to hypothesize that increased BBB permeability is directly regulated by neuronally released glutamate. However, how NMDA receptors may mediate these changes remains unclear.

In this study, we hypothesized that direct stimulation of brain endothelial cells by neurotransmitters may rapidly induce an increase in paracellular and transcellular permeability as well as increased blood flow to the activated region. We also postulated that hypofunction of the implicated receptors may be linked to the dysfunction of neurovascular coupling, potentially inducing neurodegenerative diseases. In this study, we have focused on the role of NMDA receptor signaling in the regulation of BBB permeability. Our investigations were provoked in part by the recent study by Vazana et al. [40], who observed that NMDA receptor activation can induce increased permeability from the brain side. In our study, we used in vitro and in vivo assays to investigate the mechanisms underlying an NMDA-receptor-mediated increase of BBB permeability. NMDA receptor stimulation increased Ca^2+^ influx, leading to increased localization of the clathrin heavy chain to the endothelial cell surface and the aggregation of Caveolin-1. Together, these two molecules contribute to transcytosis of brain endothelial cells. NMDA receptor stimulation also induced an increase in the quantity of early endosomes and a decreased level of lysosome trafficking. This result is consistent with an increased level of molecular delivery that is mediated through activation of CaMKII and subsequent stimulation of RhoA and PKC. In addition, NMDA application in the mouse brain transiently increased permeability of the BBB to transferrin and IgG, which are large molecules that normally barely cross the BBB. These phenomena were blocked by the co-administration of a selective antagonist of NMDA receptors, suggesting that these reactions are specific to the NMDA receptor. Experiments using specific ablation of NMDA receptors in BBB endothelial cells decreased vascularization in a time-dependent manner in knockout mice when compared to littermate controls. Moreover, the number of neurons in the brain also decreased gradually, reaching its lowest level at 8 months after birth, indicating that decreased vascularization and neuronal loss were induced by dysfunction of NMDA receptor signaling in endothelial cells. Overall, these findings suggest that NMDA receptor signaling in brain endothelial cells is critical in the regulation of BBB permeability. Furthermore, dysfunction of NMDA receptor signaling can lead to neuronal loss, emphasizing the importance of molecular delivery mediated by neurotransmitter signaling in brain endothelial cells.

## Results

### NMDA receptors expressed in human brain endothelial cells

**Fig. 1:**
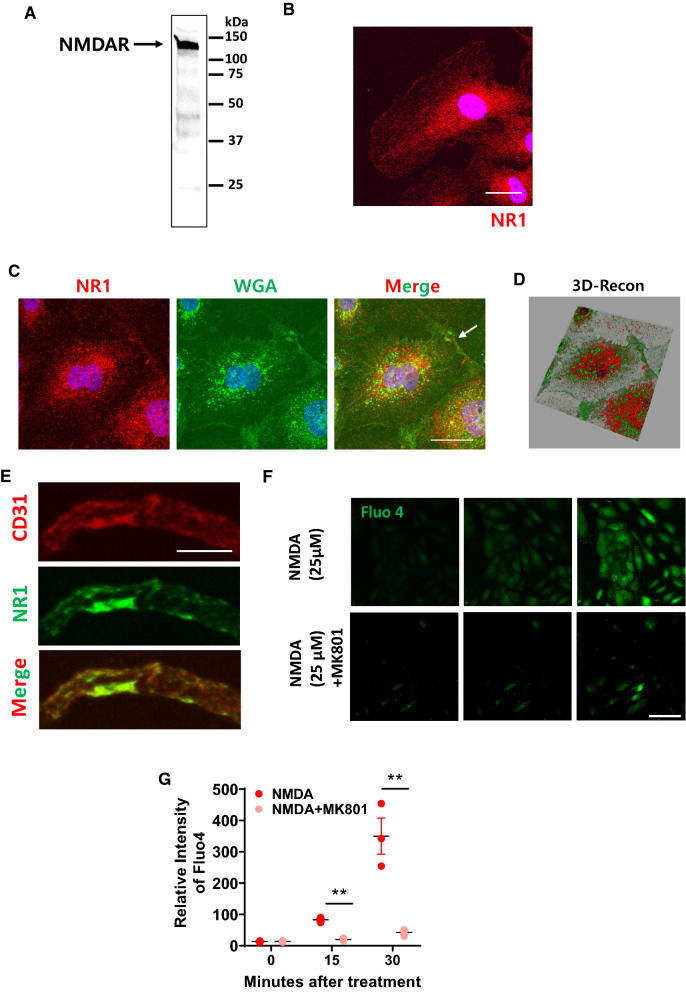
Expression of NMDA receptors in the human primary brain endothelial cells and its activation induces Ca^2+^ influx. **A** and **B** Expression of the human NMDA receptor subunit 1 (NR1) in the primary human brain endothelial cells were confirmed using western blot (**A**) and immunofluorescence assay (**B**). Scale bar indicates 25 μm. **C** and **D** (**C**) Confirmation of NR1 expression on the cell surface. Primary human brain endothelial cells were stained with anti-NR1 (Red, 1:500), wheat germ agglutin (WGA, Green, 1:1000). Arrow indicates where surface localization of NR1 is observed. **D** Image from (**C**) was 3D reconstructed by LAS X 3D visualization software. **E** Expression of NR1 on the brain endothelial cells in vivo. Brain endothelial cells were stained with CD31 (Red), NR1 with anti-NR1 (Red, 1:100). Scale bar indicates 5 μm. **F** and **G** (**F**) Application of NMDA induces influx of Ca^2+^ in the brain endothelial cells (upper panel) that is blocked with application of MK801, a specific blocker of NMDA receptor (bottom panel) that was measured by real time confocal microscopy. Scale bar indicates 75 μm. **G** Relative intensity of Fluo 4 was quantified and was presented as a graph (n = 4, two-tailed unpaired student t-test, ** indicates where p value is less than 0.01)

NMDA receptors are known to be highly expressed in neuronal cells [41, 42] and oligodendrocytes [43, 44]. In neurons, NMDA receptor activation mediates slow depolarization and regulates stimulus intensity and frequency, so we questioned whether NMDA receptor signaling in brain endothelial cells also has a physiological effect on brain endothelial cells. First, the primary brain endothelial cells were tested if they have the characteristics found in the functional brain endothelial cells. Expression of tight juction and adhesion molecules, accumulation of acetylated low density lipoproteins, and von Willebrand Factors that are characteristics of the brain endothelial cells were confirmed by immunofluorescence assay (Additional file 1: Figure S1). Of interest, accumulation of acetylated low density lipoproteins and expression of von Willebrand Factors were hetereogeneous (Additional file 1: Figure S1). We performed western blot and immunocytochemical analysis in primary cultured brain endothelial cells and confirmed that they express the NMDA receptor subunit 1 (NR1) protein (Fig. 1A and B), as has been shown previously [42, 45]. A co-localization assay using wheat germ agglutinin (WGA) as a marker for the cell surface demonstrated the localization of NR1 on the cell surface and in the cytoplasm (Fig. 1C and D). Additionally, expression of NR1 in murine brain endothelial cells on both the apical and the basolateral side was observed (Fig. 1E). This is partially in accordance with the findings of Lu et al. [45], who reported similar NR1 expression, but it was higher on the basolateral side [45]. Although the NR1 subunit is a core component of NMDA receptor channels, its presence in brain endothelial cells is not sufficient to confirm if it is present in functional ion channels that might be responding to glutamate and other chemical mediators released from nerve endings or astrocytic endfeet.

### Activation of NMDA receptors increases intracellular calcium in brain endothelial cells

If the NR1 subunit expressed by brain endothelial cells is present in functional ion channels, its activation should lead to a local increase in intracellular Ca^2+^ concentration. Figure 1C shows the results of a live calcium imaging study in cultured endothelial cells treated with 25 μM of NMDA. Ca^2+^ ion influx was tracked using Fluo4, a Ca^2+^ indicator, that was measured with time-lapse confocal microscopy. A strong influx of Ca^2+^ ions into the cytosolic space was observed that was maintained up to 15 min, indicating that the NR1 subunit in brain endothelial cells is part of functional ion channels (Fig. 1F and G). This influx was downmodulated when brain endothelial cells were incubated with the NMDA receptor inhibitor MK801, indicating that the increase in intracellular Ca^2+^ was, at least in part, due to influx through activated NMDA receptor channels (Fig. 1F and G). This is important because Ca^2+^ is an important second messenger that may trigger cellular responses in brain endothelial cells [46, 47]. This result is contrary to that of a recent publication by Mehra et al. [48] that did not observe a Ca^2+^ influx in brain endothelial cells upon administration of NMDA.

### Activation of NMDA receptors induces relocalization of transcytotic markers enhancing molecular delivery mediated by CaMKII signaling pathway

**Fig. 2:**
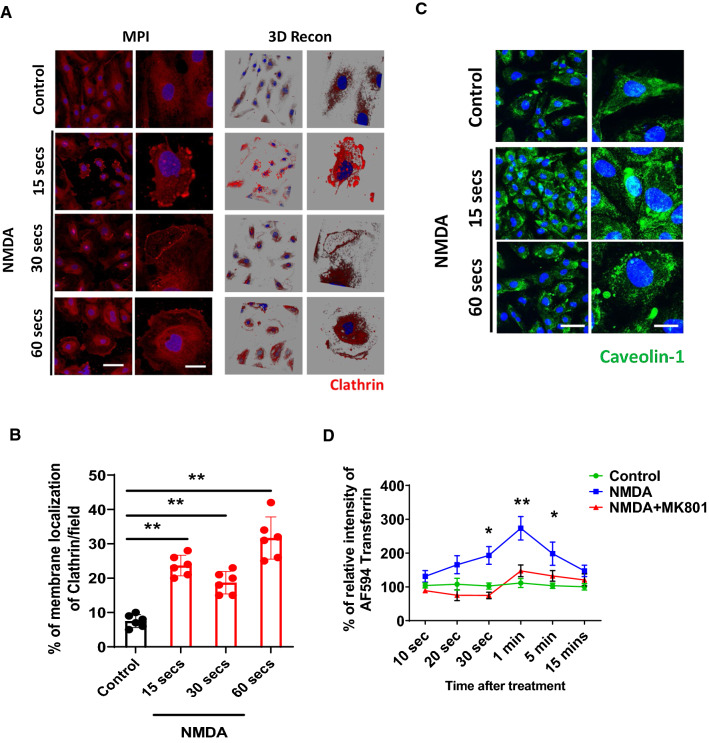
Activation of NMDA receptor induces translocation of molecules that are involved in transcytosis in bran endothelial cells. **A** Primary human brain endothelial cells were treated with NMDA (25 μM) for different time points and cells were fixed and stained with Clathrin heavy chain (Red). 3D reconstructed images were placed on the right side for better appreciation of clathrin heavy chain localization at the cellular edge. Magnified images from left panel are presented on the right panel in both. Scale bar indicates 75 μm and 25 μm for left and right panel, respectively. **B** Percentage of cells that is positive for the membrane localization of Clathrin heavy chains were quantified and depicted as a graph (n = 6, two-tailed unpaired student t-test, ** indicates where p < 0.01). **C** Activation of NMDA receptor induces redistribution of Caveolin 1 (Green). Scale bar indicates 75 μm and 25 μm. (**D**) AF594-transferrin permeability assay on the primary brain endothelial cells cultured on the porous membrane. Cells were pretreated with 10 μg/ml of AF594-transferrin with or without NMDA (25 μM) or MK801 (1 μM) and the transmigrated AF594-transferrin was measured using Fluorescence intensity of the AF594 conjugated transferrin that crossed the membrane were measured with excitation at 590 nm and emission at 617 nm with a Flex-Station 3 and its absolute concentration was calculated using standard curve. Values of transferrin concentration of treated groups were divided by those of control groups and percentage differences were presented as a graph (n = 4, two-way ANOVA, *, ** indicates where p < 0.05 and p < 0.01, respectively)

Brain endothelial cells take up different classes of molecules and deliver them transcytotically to the brain parenchyma through the formation of clathrin-coated pits or lipid rafts. The manner of transcytotic delivery used is determined by the compartment to which a molecule is delivered—either the early endosome or the lysosome [6, 7, 19, 21, 49, 50]. In order to further dissect the mechanisms behind NMDA-induced enhancement of molecular delivery, we treated primary murine brain endothelial cells with NMDA for different time intervals and stained for clathrin heavy chain and Caveolin-1, which both play major roles in transcytotic pathway [49, 51]. Upon administration of NMDA, rapid relocalization of clathrin heavy chain to the plasma membrane surface was observed. NMDA-treated brain endothelial cells formed pocket-like shapes or ruffles that colocalized with the clathrin heavy chain, possibly as a result of increased endocytosis and consequent molecular delivery (Fig. 2A). Co-treatment of brain endothelial cells with NMDA and the noncompetitive antagonist MK801 induced a decrease in clathrin relocalization (Fig. 2A and B), suggesting that this reaction is specific to the effects of NMDA receptor channel signaling. Moreover, surface localization of Caveolin-1, which is a marker for lipid rafts, and its aggregation was higher after NMDA treatment when compared to controls, suggesting that molecular delivery via transcytosis may have been enhanced by NMDA treatment (Fig. 2C). Next, an in vitro BBB functional assay showed that NMDA could enhance the receptor mediated transcytosis of AF594-Transferrin rapidly and temporarily, peaking at 1 min and returning to the basal/control level 15 min after treatment that did not induce changes in the junction formation (Fig. 2D and Additional file 1: Figure S2). This increase in BBB transcytosis was blocked by 1 μM of MK801 (Fig. 2D). Also, the increased transcytosis was shown to be concentration dependent (Additional file 1: Figure S3). Together, these results imply that NMDA treatment functionally enhances the transport of molecules via receptor-mediated transcytosis. This supports the hypothesis that NMDA signaling contributes to regulating transcytosis of molecules through the BBB. While an NMDA-mediated increase in BBB permeability has been reported previously [35], our data show that the opening of the BBB is much more brief. This indicates that barrier opening is transient, implying tight regulation imposed by the brain itself in order to maintain its normal physiology.

**Fig. 3:**
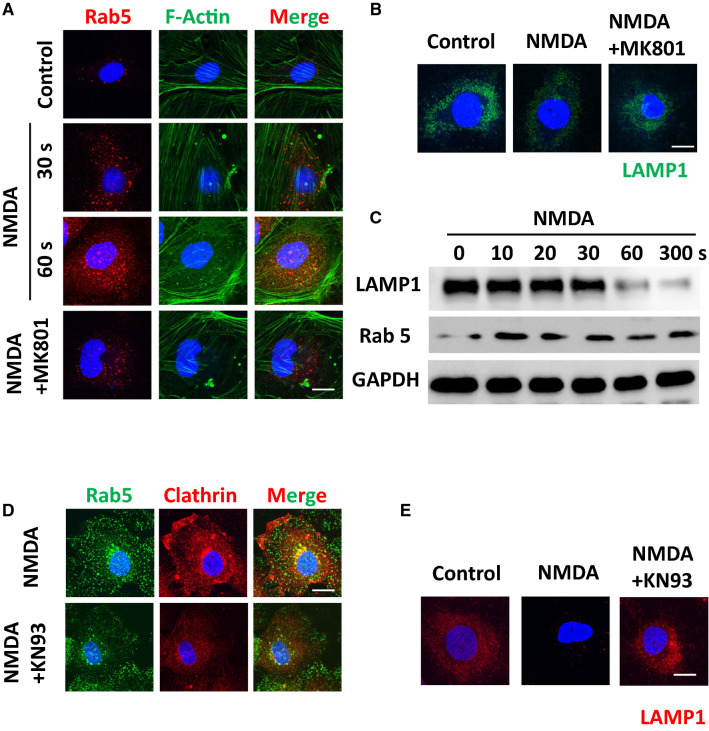
Activation of NMDA receptor induces increase in the early endosomal trafficking pathway and suppression in the lysosomal trafficking pathway mediated through CaMKII activation. **A** NMDA receptor activation (NMDA 25 μM) causes increases in the Rab5 (Red), a marker for the early endosome that was blocked by MK801 (1 μM). Cells were counter stained with phalloidin for actin staining (Green). Scale bar indicates 25 μm. **B** Activation of NMDA receptor (NMDA 25 μM) induces suppression of LAMP1 (Green), that was recovered with the treatment of MK801 (1 μM). Scale bar indicates 25 μm. **C** Western blot analysis of Rab 5 and LAMP1 show rapid upregulation of Rab 5 and down modulation in LAMP1. GAPDH was used for loading control. **D**–**E** Stimulation of NMDA receptor induces activation of CaMK II. **D** Suppression of CAMKII activity decreases level of early endosome and reverses surface re-localization of clathrin heavy chain triggered by NMDA receptor stimulation. CaMKII activity was suppressed with pretreatment of KN93 (2.5 μM, 15 min) and further treated with NMDA (25 μM) for 30 s. Cells were stained for Rab 5 (Green) and Clathrin (Red). Scale bar indicates 25 μm. **E** Suppression of CaMKII activity reverses the decreased LAMP1 positive signal caused by activation of NMDA receptor in the brain endothelial cells. CaMKII activity was suppressed with pretreatment of KN93 (2.5 μM, 15 min) further treated with NMDA (25 μM) for 30 s. Cells were fixed and further stained with LAMP 1 (Red). Scale bar indicates 25 μm

Recent studies have shown that brain endothelial cells selectively regulate the delivery of molecules into the brain through modulation of the endosomal trafficking pathway [7, 50]. To test if NMDA receptor activation can induce activation of endosomal trafficking pathways and downmodulate the lysosomal pathway, we treated brain endothelial cells with NMDA and measured the abundance of early endosomes and lysosomes using their respective specific markers, Rab5 and LAMP1. We observed that the level of early endosomes rapidly increased for up to 1 min upon NMDA administration, and that this effect was suppressed by co-treatment with MK801 (1 μM), indicating that NMDA channel activation can specifically enhance the early endosomal trafficking pathway (Fig. 3A). Brain endothelial cells utilize the late-endosomal-lysosomal pathway to degrade molecules and limit their entry into the brain. We tested whether NMDA receptor signaling may suppress this pathway to better enhance molecular delivery into the brain [7, 19]. Indeed, induced suppression of LAMP1 positive signal upon NMDA treatment was found, which was reversed by MK801, supporting our hypothesis that the late-endosomal-lysosomal pathway is suppressed by NMDA-mediated signaling (Fig. 3B). These time-dependent changes were confirmed by western blot analysis, which also showed that NMDA receptor activation can enhance the early endosomal pathway while downmodulating the lysosomal pathway. Together, these findings indicate that NMDA receptor signaling in brain endothelial cells enhances transcytotic molecular delivery via a convergence of effects on key trafficking mechanisms (Fig. 3C). We have observed that the transcytosis of brain endothelial cells increases upon activation of NMDA receptor signaling. We next decided to dissect the molecular mechanisms behind this phenomenon. It is widely accepted that the signaling cascade of NMDA receptor activation in neuronal cells is mediated by CaMKII and its downstream pathways [30, 52]. First, to test if NMDA receptor signaling can enhance the phosphorylation of CaMKII, we used western blot analysis and immunofluorescence assays to test the effect of NMDA receptor activation on the phosphorylation status of CaMKII. An increase in the phosphorylation of CaMKII at a very early time point after activation of NMDA receptor was observed. Next, we co-administered 2.5 μM of KN93, which is an inhibitor of CaMKII phosphorylation, and after 15 min observed an increase in Rab5 localization and inhibited membrane relocalization of clathrin. This indicates that CaMKII activation is the major signaling cascade that enhancing delivery of molecules to the CNS by NMDA receptor signaling.

### NMDA-activation-mediated transcytosis is induced by activation of RhoA and Protein Kinase C

**Fig. 4:**
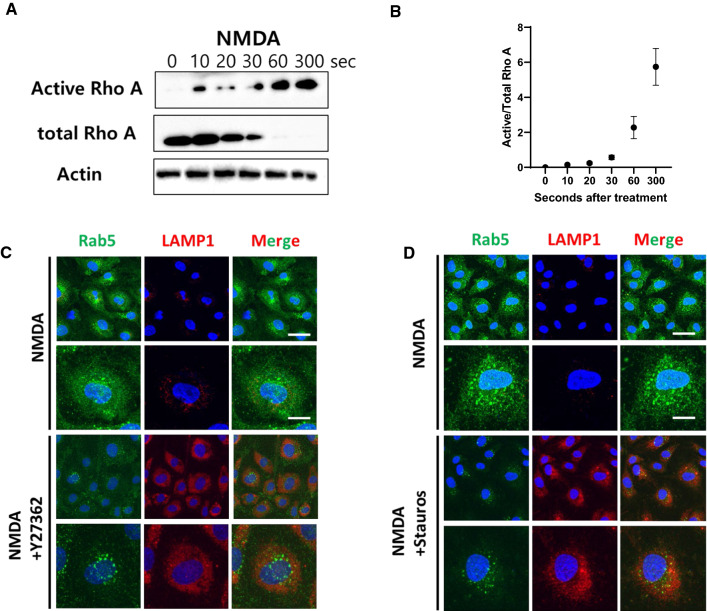
Activation of NMDA receptor induces rapid modulation of Rho-GTPase and actin cytoskeletal reorganization (**A**) Activation of NMDA receptor induces upregulation of the Rho-GTPase activity and was depicted as a graph (n = 3). **C** Inhibition of Rho associated kinase by Y27362 (0.5 μM) in NMDA treated brain endothelial cells decreases in Rab5 (Green), and enhances LAMP1 (Red). Scale bar indicates 75 and 25 μm for upper and bottom panel, respectively. Images from upper panel is enlarged and presented at the bottom panel. **D** Inhibition of PKC by Staurosporine (2 nM) induces decreases in Rab5 (Green) and enhances LAMP1 (Red) compared to its NMDA only control. Images from upper panel is enlarged and presented at the bottom panel. Scale bar indicates 75 and 25 μm for upper and bottom panel, respectively

Several lines of study suggest that actin-cytoskeletal reorganization is the master signaling pathway governing endosomal trafficking [53]. Rho GTPase is a master regulator of the actin cytoskeleton. Since RhoA is a molecule in a pathway downstream of CaMKII, we hypothesized that CaMKII-mediated regulation of endosomal trafficking may be promoted by RhoA signaling [54, 55]. To test if NMDA-receptor-mediated stimulation can induce RhoA activation, we administered NMDA and then performed a Rho-GTPase activity test based on a pull-down assay of the active form of RhoA using the Rho-binding domain (RBD), which specifically binds to the active form of RhoA. Active form of RhoA increased beginning from 10 s after NMDA treatment, and that this increase was maintained for up to 5 min (Fig. 4A and B), suggesting that Rho GTPase activity is upregulated by NMDA receptor signaling.

Since we observed an increased signal of early endosome upon treatment with NMDA, we wondered if such increased early endosomal markers in brain endothelial cells could be affected by RhoA signaling. To test this, we administered Y27362, a specific inhibitor for Rho-associated kinases, to test the effect of RhoA downstream signaling on endosomes in brain endothelial cells. A decrease in early endosome abundance was observed, and a decreased level of LAMP1 was recovered 30 min after treatment with 0.5-μM Y27362. This indicates that RhoA signaling is important in NMDA-dependent enhancement of transcytosis (Fig. 4C). Protein kinase C (PKC) is a downstream effector molecule of CAMKII that regulates endosomal trafficking in neuronal cells [55, 56]. To test if CAMKII activation can indeed increase the activity of PKC and thereby regulate endosomal trafficking, we suppressed the activity of PKC with 2-nM staurosporine, which is a nonselective inhibitor of PKC, for 30 min. We then performed an immunofluorescence assay to observe Rab5 localization and the formation of lysosomes in brain endothelial cells. As a result, increases in Rab5 levels upon NMDA stimulation was downmodulated through inhibition of PKC activity. Additionally, the depression of lysosomal formation by NMDA stimulation was recovered through PKC inhibition, indicating that PKC signaling is the major signaling cascade inducing lysosomal depression in brain endothelial cells (Fig. 4D). Overall, we conclude that increased transcytotic molecular delivery in brain endothelial cells upon NMDA stimulation is mediated in a RhoA- and PKC-dependent manner.

### Activation of NMDA receptors induces rapid opening of the BBB through activation of transcytotic pathways in the mouse brain

**Fig. 5:**
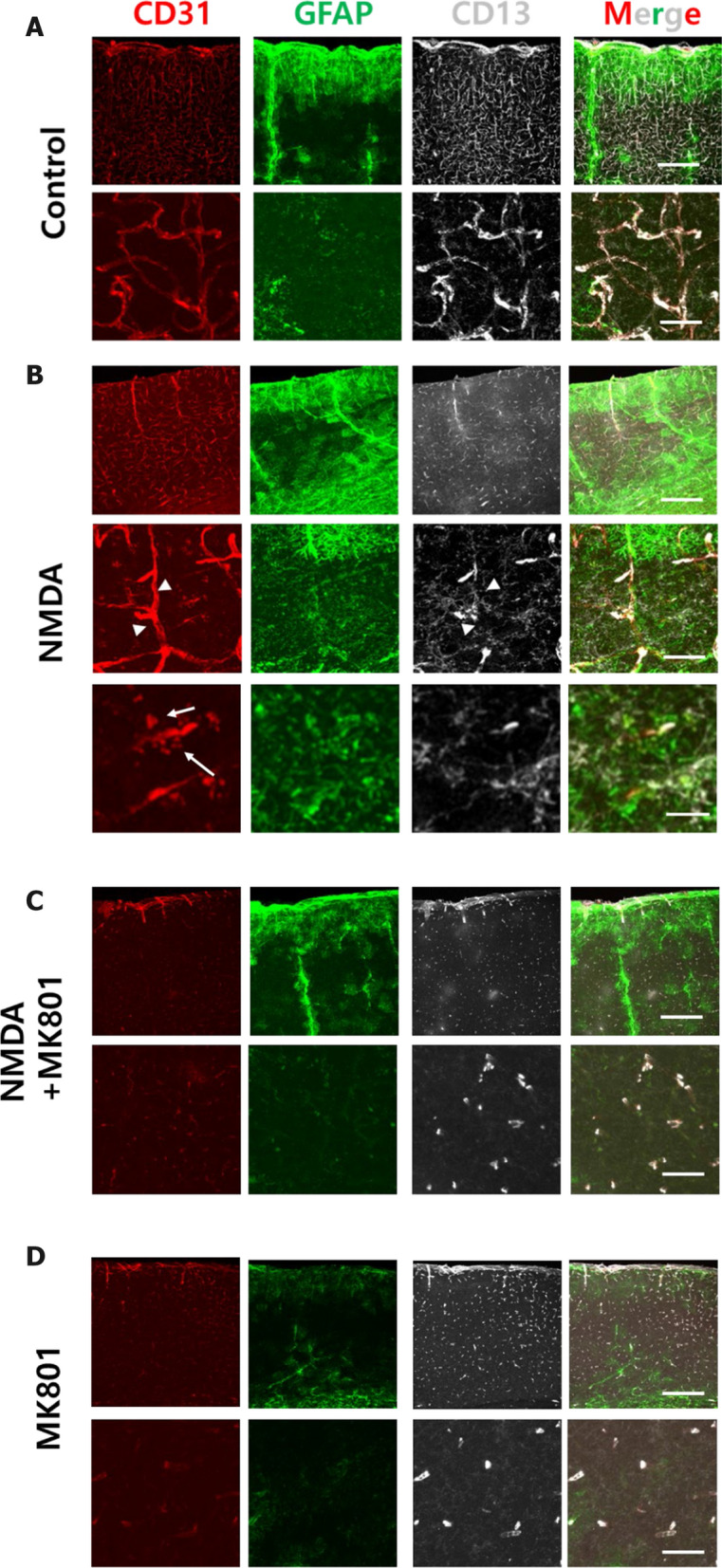
Activation of NMDA receptor induces rapid dislocalization of markers for the BBB at the capillary level in the mouse cortex. **A**–**D** Activation of NMDA receptor induces rearrangement of the brain endothelial cells and pericytes in a receptor specific manner. C57/BL6 mouse was injected with PBS (**A**), NMDA (0.5 mg/kg) (**B**), NMDA with MK801 (0.5 mg/kg each) (**C**), and MK801 (0.5 mg/kg) (**D**) for 1 min and mice were sacrificed. Brain sections were stained with CD31 (Red, 1:500), GFAP (Green, 1:500), and CD13 (Gray, 1:500), a marker for endothelial cells, astrocytes, and pericytes, respectively. Magnified images of each treatment are displayed at the bottom panel. Arrow indicates the CD31 positive lamellipodia like structure. Arrowhead indicates where devoid of CD13 signal is observed at the brain vasculature is observed. Scale bar indicates 200 μm and 25 μm for upper and bottom panel, respectively. For NMDA treated mouse (B) Scale bar indicates, 200 μm (top panel), 25 μm (middle panel), 5 μm (bottom panel)

Having observed that NMDA receptor signaling can increase transcytosis of peripheral molecules through human brain endothelial cells in vitro, we wondered whether NMDA receptor signaling could enhance barrier permeability by directly acting on brain endothelial cells in vivo. Since the effects of NMDA receptor activation which led to BBB opening in vitro were linked to components of transcytotic pathways, we subsequently questioned if a similar permeabilization pattern would be observed in the mouse brain in vivo. Because the brain parenchyma is constantly activated with neurotransmitters, it is very hard to distinguish the effect of externally applied NMDA from that of glutamate released by neuronal activity in the brain. In addition, direct injection of molecules into the brain parenchyma naturally induces damage to the brain vasculature, which would compromise the interpretation of the effect of NMDA on brain endothelial cells. To overcome these limitations, we decided to directly inject NMDA into the periphery via the retro-orbital route and visualized the integrity of the BBB using several component markers including CD31, CD13, and GFAP, which are markers for endothelial cells, pericytes, and astrocytes, respectively. First, the structural changes of the BBB in the cortical region were analyzed. It was found that intensity of CD31 increased globally after NMDA injection, and CD31 positive signals were observed outside of cortical vascular regions (Fig. 5 A and B). However, it did not induce the infiltration of peripheral immune cells in the brain parenchyma suggesting that the peripheral cellular infiltration may not be the inducing factor for the increased CD31 (Additional file 1: Figure S4A). Also, activation of adhesion molecule is specific for CD31 since ICAM-1 expression was not increased upon NMDA treatment (Additional file 1: Figure S4B). The pericyte marker CD13 showed dissociation from the CD31-positive vascular region, indicating that NMDA receptor signaling can potently induce the dissociation of these cells from their close association with endothelial cells in an intact BBB. Interestingly, astrocytes were more closely recruited to the vicinity of the vascular region after NMDA treatment (Fig. 5A and B). Co-treatment of NMDA and MK801 showed shrinkage of the vasculature compared to NMDA alone or the vehicle control (Fig. 5C). The dissociation of pericytes from the vascular region in the co-treated NMDA and MK801 group was similar to that observed in the NMDA-only treatment group, which may be attributable to the partial agonistic effect of NMDA (Fig. 5D). Alternatively, it could be attributable to an effect of NMDA on pericytes that is distinct from NMDA activation of ion channels, since this phenomenon was similarly maintained in the MK801-only treatment group, where the signal of CD31 was barely observed. Together, these experiments indicate that blockade of the NMDA receptor-channel by MK801 can prevent vasculature dilation at the capillary level, suggesting that it is triggered by Ca^2+^ entry (Fig. 5D). Furthermore, dissociation of the cellular components of the BBB is mediated by NMDA receptor activation, and its blockade can decrease the diameter of capillaries in the brain. We subsequently explored if these phenomena are universally observed throughout the brain. It is shown that the activation of the NMDA receptor in the hippocampus induce less changes in the cellular component in the neurovascular unit (intensity of CD31, dissociation of pericytes from brain endothelial cells, activation of astrocytes) whereas its inhibition induced similar results as it did in the cortex (Additional file 1: Figure S5). In the ventral striatum, NMDA treatment decreased CD31 and dissociation of CD13 whereas blockade of NMDA receptors induced general decrease of CD31, CD13, and GFAP levels (Additional file 1: Figure S6). This indicates that the NMDA receptor activation results in different patterns of responses in the components of the NVU, showing the heterogeneity of responses.

**Fig. 6:**
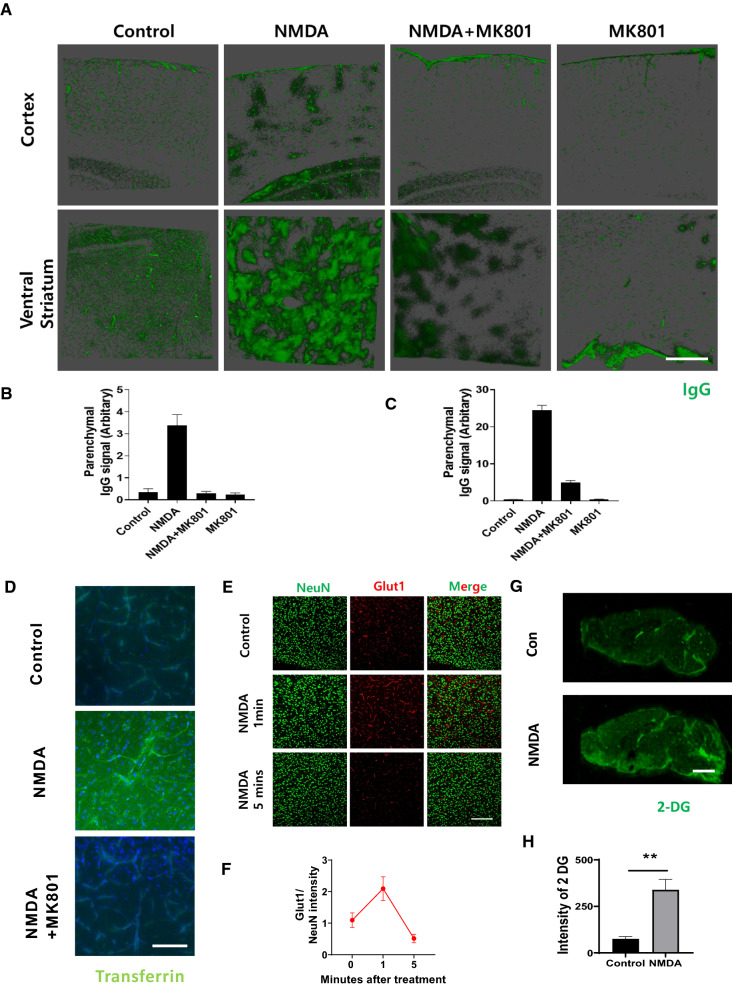
Activation of NMDA receptor induces increased permeability of the BBB to various bioactive materials at the capillary level. **A** Activation of the NMDA receptor induces increased permeability to the immunoglobulin from peripheral system. C57/BL6 mouse was injected retro-orbitally with PBS, NMDA, NMDA with MK801, and MK801 for 1 min. Brain sections were stained with IgG (Green) to visualize the permeability upon its activation. Scale bar indicates 500 μm. **B** and **C** Signals of IgG in the parenchymal area for cortex (**B**) and ventral striatum (**C**) were measured and quantified by subtracting the total signal of IgG in the vascular area by that of vascular area and depicted as a graph (3 different area each from 3 different animals). **D** NMDA induces increased accumulation of Transferrin (Green), a marker for the representative transcytosis through the BBB. Scale bar indicates 75 μm. (**E**–**H**) Injection of the NMDA induces rapid upregulation of Glut1 and increased permeability to the fluorescently labeled 2-deoxyglucose (2-DG). **E** NMDA was injected brain sections were stained for Glucose transporter 1 (Glut1, Red). NeuN was counterstained for neuronal cell (Green). **F** Values of Glut 1 was divided by that of NeuN and the values were depicted as a graph (n = 4). Scale bar indicates 200 μm. **G** 10 nmol of near infrared (NIR)-2-DG with or without the NMDA was injected. Brain was sectioned and the accumulation of 2-DG (Green) in the brain was scanned with IRDye scanner. Scale bar indicates 2 mm. **H** Intensity of NIR-2-DG from (**G**) was quantified and depicted as a graph (n = 3, two-tailed unpaired student t-test, ** indicates where p < 0.01)

Since we observed changes in localiztion of molecules involved in transcytosis and structural changes including lamellipodia formation in the brain endothelial cells and dissociation of pericytes from endothelial cells in the in vivo study, which may correlate with increased delivery of molecules in our in vitro model of BBB. Therefore, we next tested whether NMDA receptor signaling can also induce increased delivery of molecules in vivo, again using the retro-orbital route for NMDA and MK801 injections. We chose to investigate the effect of NMDA on IgG transcytosis across the BBB, which is a peripheral molecule that does not pass the BBB under normal physiological conditions. As shown in Fig. 6, the transcytosis of IgG increased dramatically in the cortex and the ventral striatum after NMDA injection when compared to control. Together, these experiments strongly implicate a role for NMDA receptor signaling in the regulation of BBB permeability (Fig. 6A–C). The increased transcytosis of IgG through BBB upon NMDA activation was maintained up to 5 min that returns to its basal level at 15 min (Additional file 1: Figure S7). Further, increased transcytosis of IgG at the hippocampus was not as dramatic as that of the cortex and ventral striatum, indicating the regional specificity of this phenomena (Additional file 1: Figure S8). In order to test our hypothesis further, we decided to determine if NMDA receptor signaling can enhance the delivery of bioactive molecules that can be used in neuronal cells. Transferrin is one of the most abundantly transported molecules to the brain via transcytotic pathways [14]. As a result, increased transport of transferrin after NMDA injection was observed. Higher transferrin signal was detected in the brain parenchyma and perivascular area of the brain vasculature when compared to control (Fig. 6D). Again, importantly, increased transferrin transport was downregulated when MK801 was co-administered with NMDA, indicating that this response is also NMDA-receptor-specific (Fig. 6D).

Lastly, we investigated whether NMDA receptor signaling influences glucose entry into the brain, since glucose is the most important energy source for neuronal health. After NMDA treatment, the expression level of the brain glucose transporter GLUT1 increased within 1 min; this increase was again transient, decreasing to near normal levels 5 min after treatment (Fig. 6E and F). This suggests that glucose transporter expression levels are regulated by NMDA in vivo via the regulation of endothelial transporters to increase the BBB’s transportation of molecules essential to proper brain function. Consequently, we questioned whether NMDA receptor channel activity indeed increases the delivery of glucose across the BBB in a way that can enhance its entry into the brain. For this, NIR-2DG, which is fluorescently labeled 2-deoxyglucose and is a non-hydrolyzable analog of glucose, was used to track glucose accumulation in the brain. We found that NIR-2DG accumulation in the brain after NMDA treatment was significantly higher when compared to untreated controls. This suggests that NMDA receptor activation by synaptically released glutamate can facilitate the transport of glucose to the brain (Fig. 6G and H). Overall, our study demonstrates that molecular delivery through the BBB is increased by NMDA receptor signaling, thereby enhancing the molecular delivery of some required large molecules and bioactive molecules into the brain.

### Lack of endothelial NMDA receptors leads to a decrease in brain vasculature in a time-dependent manner, followed by decreased numbers of neurons

**Fig. 7:**
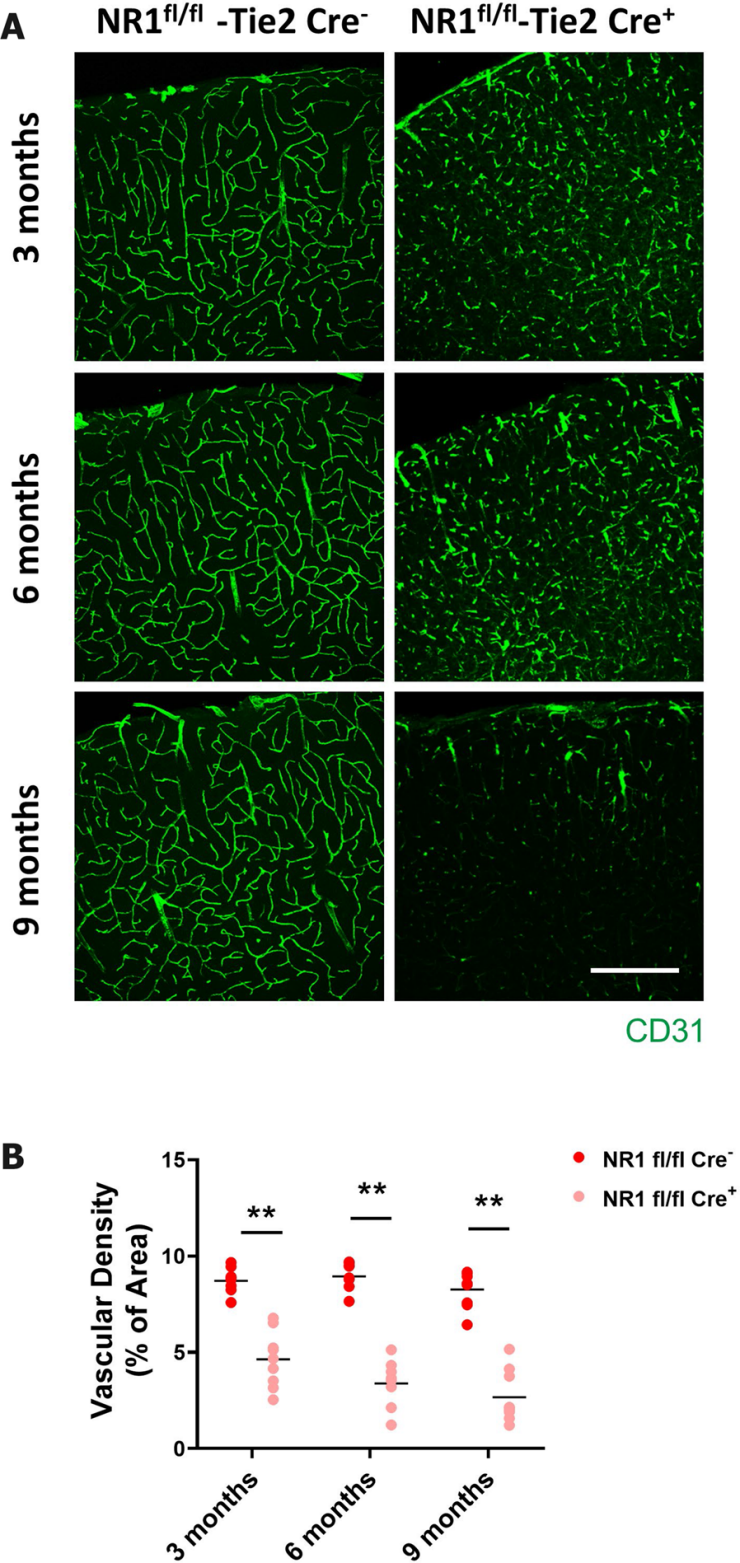
Ablation of the NMDA receptor in endothelial cells induces decrease in the brain vasculature. **A** and **B** (**A**) Brains from the 3, 6, and 9 months old endothelial cell specific NMDA receptor subunit 1 (NR1) knock out (NR1^fl/fl^-Tie2Cre^+^) and its littermate control (NR1^fl/fl^-Tie2Cre^−^) was stained with CD31 a marker for the brain endothelial cells. Scale bar indicates 200 μm. **B** Density of brain vasculature (percentage of brain area) was quantified by Image J and depicted as graphs (** indicates where p < 0.01, two-tailed unpaired student t-test, 3 different area each from 3 different animals)

Given that NMDA receptor signaling appears to regulate molecular delivery across the BBB in vitro and in vivo, we questioned if genetic modification of NMDA receptor genes, specifically at the level of endothelial cells, can influence functional and/or morphological changes in the blood vessel, and possibly also subsequently affect neuronal survival. As a means to test whether NMDA receptor signaling indeed induces these types of changes, we designed a conditional knockout mouse of NMDA receptor subtype 1 (NR1), which is specific to endothelial cells, by crossing floxed NR1 mice with Tie2-cre mice, thus incorporating Cre under Tie2-promoter specific for endothelial cells [57]. Since NR1 is a major component of NMDA receptor channel complexes, we reasoned that knocking out NR1 from cerebral vasculature would result in a loss of NMDA-receptor-mediated control of BBB permeability. Even though general knockout of the NR1 induces a lethal phenotype, vasculature-specific NR1 knockout mice did not show significant deficits, indicating that the NMDA receptor is not a core component at the developmental stage we used. We next investigated two potentially interrelated questions. First, we asked whether NR1 deletion from cerebral vasculature affects the BBB in vivo. Clearly, the NR1 knockout mouse had less vascularity, branching, and tortuosity in the brain beginning from 3 months after birth when the thickness of the brain vasculature was significantly less than that of littermate controls (Fig. 7A and B). This suggests that NMDA receptor signaling in brain endothelial cells is important in maintaining the brain vasculature as well as in regulating its diameter, which is important for proper blood flow.

**Fig. 8:**
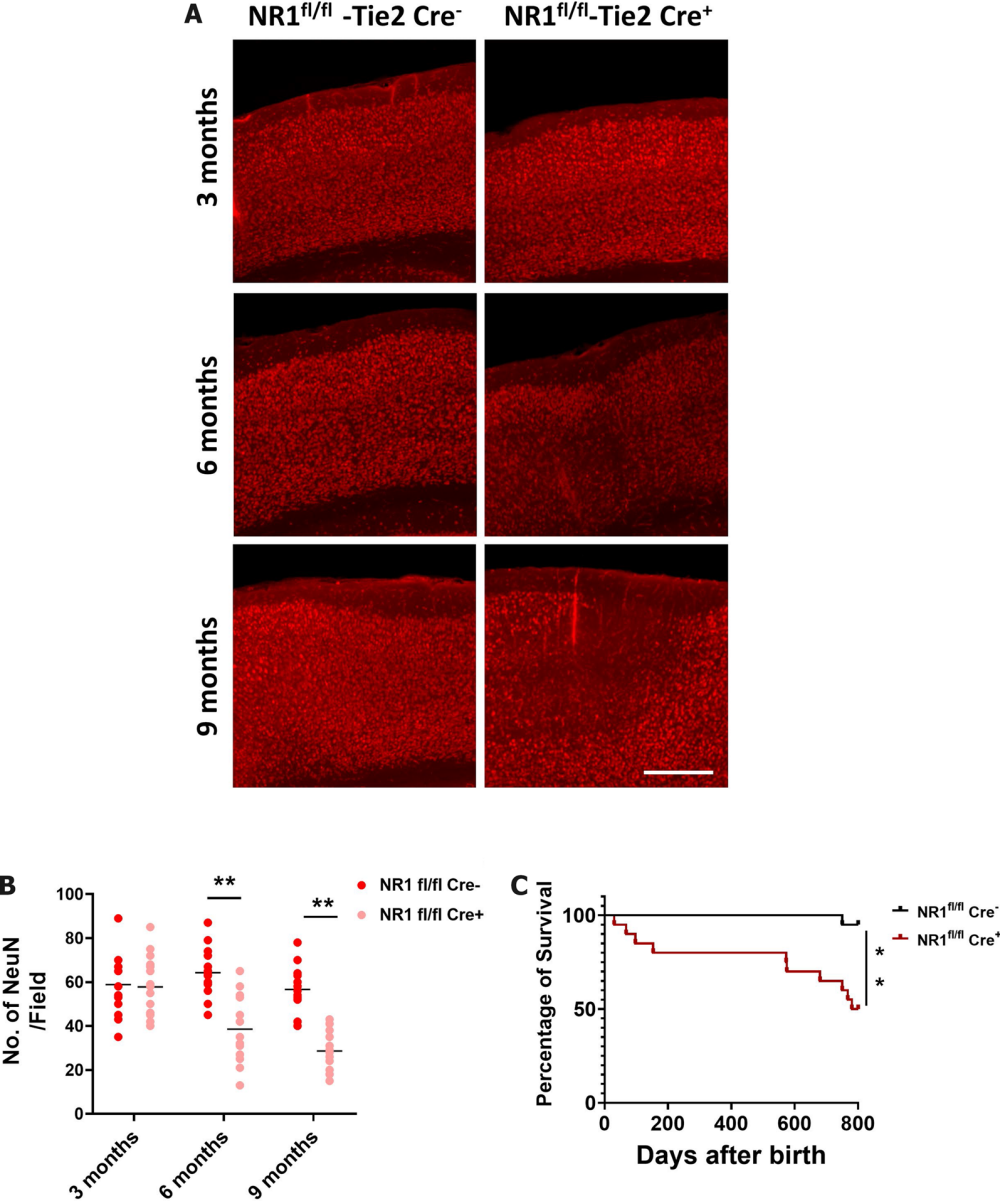
Ablation of the NMDA receptor in the endothelial cells induces decreased level of neuronal cells in the brain and its survival is decreased. **A** Brains from the 3, 6, and 9 months old endothelial cell specific NR1 knock out (NR1^fl/fl^-Tie2Cre^+^) and its littermate control ((NR1^fl/fl^-Tie2Cre^−^) were fixed and sectioned to stain with NeuN (Red) a marker for neuronal cells. Scale bar indicates 400 μm. **B** Number of NeuN positive signals from cortices was quantified and depicted as a graph. (two-tailed unpaired student t-test, ** indicates where p < 0.01, 5 different area each from 3 different animals). **C** Survival rate of endothelial cell specific NR1 knockout and its littermate control was compared and depicted as a graph (** indicates where p < 0.01, Log-rank (Mantel-Cox) test)

Our second question asked how NR1 knockout, which would deplete brain endothelial cells of proper NMDA receptor channel function, would affect neuronal survival. Clearly, ablation of NR1 from brain endothelial cells would affect the molecular delivery of blood nutrients essential for the maintenance of neuronal cells. However, we wondered whether compensatory mechanisms may exist to improve neuronal survival. To address this question, we examined brain neuronal cell numbers by staining them with NeuN, which is a specific marker for neuronal nuclei, and observed that the number of neuronal cells began to decrease beginning from 6 months after birth, which is 3 months later than the observed vasculature reorganization (Fig. 7A and B, Fig. 8A and B). This observation supports our hypothesis that lack of sufficient nutrient delivery leads to neurological disfunction. Additionally, lack of NR1 in brain endothelial cells correlated with decreased longevity, suggesting that decreased neuronal function led to premature death of the knockout mice (Fig. 7C). Overall, these findings suggest that NMDA receptor signaling in the brain vasculature is crucial for maintaining functional molecular delivery of essential nutrients to the brain, and that absence of brain endothelial cell NMDA receptors can be a detriment to neuronal population maintenance, either through decreased neuronal survival or decreased differentiation of neural precursors into mature neurons.

## Discussion

Molecular delivery to the brain through the inherently highly resistant vascular barrier that is the BBB has been the main area of research in studies on how to improve drug delivery for the treatment of brain diseases [58, 59]. The brain should receive nutrients from the peripheral system that pass through the BBB in a very selective manner, and finding the signaling cascade that governs this sophisticated controlling system will provide a key to understanding the mechanisms behind the mystery of the opening and closing of the BBB [11, 50]. To address this, we hypothesized that NMDA receptor signaling, which is the most widely studied neurotransmitter receptor signaling pathway, may directly regulate the BBB permeability [26, 42]. Recent studies have revealed that the NMDA receptor signaling in the brain vasculature. However, they had several pitfalls. One study was limited since researchers used direct application of NMDA receptor blockers or excessive glutamate to the brain side causing damage to brain vasculature [40]. Moreover, another study mainly focused on changes in the blood flow in the arteriole and changes in its diameter rather than on the changes in the delivery of the molecules [45, 60]. We had different point of views on the reaction by NMDA receptor activation on the brain vasculature that it may cause enhanced molecular delivery rather than affecting its diameter. To address this matter and overcome several pitfalls of previous studies, we mainly focused on the mode of action of molecular delivery induced by NMDA receptor activation using an in vitro BBB model for its molecular dissection. Also, we used an in vivo model with intravenously administered NMDA to dissect changes in the structure and function of the BBB. Moreover, we used a conditional knockout model system to observe the effect of NMDA receptor signaling on the integrity of the brain vasculature.

This study shows that NMDA receptor activation can induce a potent increase in the Ca^2+^ influx. Since NMDA does not exist in the naturally in the brain, we can only similarly compare the concentration of glutamate in the brain tissue to adequately speculate the concentration at the brain endothelial cells in vivo. This result is in contrast to a previous report that did not show an influx of Ca^2+^ in brain endothelial cells upon stimulation with NMDA. In that study, researchers used HCMEC/D3 cells that may have lost the properties of primary cells, meaning that the characteristics of NMDA receptors may have been changed from ionotropic to metabotropic. This discrepancy should be reassessed in future studies. The concentration of glutamate in the cerebrospinal fluids (CSF) and interstitial fluid (ISF) is approximately 4.25 μM and 2 μM [61, 62]. However, the concentration of glutamate at the synaptic terminal reaches upto 200 mM and if glutamate is released upon neuronal activation, local concentration of glutamate would be much higher than that of CSF or ISF justifying the concentration of NMDA that was used in our study [63]. Although it is not possible to compare the concentration of glutamate to that of NMDA, it is the best comparison that we can make since NMDA is not endogenous molecule. NMDA receptor activation induced increased translocation of clathrin heavy chain and Caveolin-1 to the cell surface that enhanced the uptake of FITC-dextran or AF594-conjugated transferrin in brain endothelial cells. These results suggest that the activation of NMDA receptors can open the BBB through a transcellular pathway. The opening of the barrier was rapid and reversible, and that the level of delivery of AF594-transferrin peak at 1 min and dropped back to a normal level at 15 min post-treatment (Fig. 2 D and Additional file 1: Figure S3). This is contrary to the experiments from those of Sharp et al.[35] and Mehra [48] that showed the increased permeability at later time points in the brain endothelial cells. Also, the concentration that they used was 100–1000 μM which is higher than ours. To further dissect the mechanisms behind increased transcellular permeability, we focused on the transcytotic pathways that is regulated by sophisticated endosomal trafficking pathway in the brain endothelial cells. Recent studies have shown that brain endothelial cells regulate molecular delivery through modulation of the endosomal trafficking pathway, and that molecules that are not required for the brain physiology are rapidly moved to the lysosomal degradation pathway; these major regulatory machineries for molecular delivery in the brain endothelial cells [7, 17]. After NMDA treatment, a rapid increase in early endosomal pathway that also downmodulated the lysosomal pathway was observed. This suggests that NMDA receptor activation can enhance molecular delivery by skewing activation of the early endosomal pathway. We questioned the existence of a molecular pathway that regulates NMDA receptor signaling resulting in activation of the early endosomal pathway. It is widely known that NMDA activation mediates activation of CAMKII, which in turn mediates various types of physiological reactions in neuronal cells, especially redistribution of endosomal trafficking to induce LTP [30, 52]. We observed that NMDA receptor activation could induce the phosphorylation of CaMKII, suggesting that CaMKII activity is also upregulated, and thus further suggesting a similarity in its activation mechanism to that of neuronal cells. Further chemical inhibition of CaMKII-induced suppression of the early endosome and lysosome recovery when compared to NMDA-treated HBMVECs suggests activation of the early endosomal trafficking pathway. Two of its downstream molecules, RhoA and PKC, were also shown to regulate these pathways suggesting that CaMKII-dependent RhoA and PKC may comprise the major signaling pathway enhancing the NMDA-dependent molecular delivery through transcytotic pathways.

Consistent with our findings in vitro, the increased delivery of large and bioactive molecules across the BBB following intravenous administration of NMDA was observed. After intravenous injection of NMDA, changes in the cellular structure of the BBB was observed that induced delocalization of pericytes from the endothelial cell lining, and these changes were blocked by co-administration of MK801. Also, administration of NMDA could increase delivery of IgG through the BBB, a molecule that does not normally cross the BBB in physiological conditions. Moreover, the delivery of transferrin, a molecule that is utilized to deliver iron, to the brain increased upon NMDA treatment. These two molecules are representative molecules that utilize transcytosis for their delivery to the brain, and our results highly suggest that the activation of the NMDA receptor in brain endothelial cells increases molecular delivery via transcytotic pathways.

Interestingly, decreased intensity of CD31 staining and coverages of pericyte marker CD13, by an NMDA receptor antagonist MK801 was observed. Since we did not use a fluorescently tagged animal model to track the morphological changes of the cellular components, we cannot ascertain whether these cellular components indeed decreased by the MK801 treatment. Especially considering the relatively early time point of this effect, it is very surprising. We hypothesized that the blockade of NMDA receptor signaling may have decreased level of CD31 due to a blockade of the endosomal trafficking pathway. It was observed that the blockade of NMDA receptor signaling can induce decreased staining patterns by lectin and alcian blue staining. This potentially indicates that the blockade of NMDA receptor signaling may induce down modulation of glycocalyx (Additional file 1: Figure S9A and B). Possibly, endothelial cell-derived factors affected localization of CD13 near the endothelial cells. This may occur either indirectly through soluble factors or through direct contact. As this was not previously reported this hypothetic mechanism should be clarified in future studies.

Glucose is an essential nutrient for physiological brain maintenance, and survival depends on a constant supply of glucose to the brain from the peripheral side since the brain does not store glucose. Our study showed that brain endothelial cells with activated NMDA receptor signaling showed increased delivery of NIR-2DG, a fluorescently labeled non-hydrolyzable glucose analog, indicating that NMDA receptor activation is related to the increased delivery of glucose. This is the reminiscent of the study from Saab et al. [64], who revealed the importance of NMDA-receptor-mediated glucose transporter upregulation [64]. Since the brain utilizes glucose as an energy source, the increased levels of GLUT1 and concomitant increased influx of glucose upon stimulation of the brain endothelial cell NMDA receptor. This suggests the importance of NMDA pathway in terms of energy delivery to the brain. However, The molecular signaling mechanism are not clear and should be further investigated.

Decrease in the coverage of brain vasculature in a conditional knockout model of NMDA receptor in endothelial cells also suggests that NMDA receptor signaling is essential in maintaining the vascular structure of the brain. This decrease began as early as 3 months, and was consistently observed at later time points. The most important and key feature of this study is the decrease in the number of neuronal cells in the brain at later life stages. The number of neuronal cells decline beginning at 6 months until the age of 9 months, indicating that dysfunction of the brain vasculature can affect the neuronal survival. These phenotypes were linked with decreased survival rate in knockout mice compared to their littermate controls. Based on our speculation, we hypothesize that suppression of endothelial NMDA receptor result in decreased neurovascular coupling that may have hampered the delivery of molecules required for the survival of neuronal cells. This is supported by our in vitro and in vivo data. Future study should investigate how vascular functional defects induced by ablation of NMDA affect neuronal survival. Also, as the number of neuronal cells decline, it would be interesting to monitor neurofilament light chain in plasma during the cours of this study. Also, this result would provide a hint that vascular dysfunction may be a factor that induces neurodegenerative disease. Neurodegenerative diseases are a tremendous burden for human beings, and revealing the mechanisms behind their development is one of greatest hurdles for the next generation [65, 66]. However, it is painstaking to identify causative agents, and many lines of study end in frustration. This indicates that the factors contributing to neurodegenerative diseases may be influenced by multiple, rather than a single, factor [67]. Our results show that defects in neurotransmitter signaling on the vascular side induces the neuronal loss. Therefore, loss of neurovascular coupling may be a triggering factor for the development of neurodegenerative diseases. This study clearly showed the importance of the vascular NMDA receptor signaling pathway in the delivery of molecules to the brain. This strongly suggests that neurotransmitter-mediated activation of brain endothelial cells can enhance nutrient delivery to the brain. This signal could be an endogenous modulating signal that the brain uses to enhance the delivery of molecules through the BBB to the brain side. Exploring the underlying mechanism of brain endothelial NMDA signaling may aid in enhancing the delivery of therapeutics to the brain. The BBB is a biological barrier that protects the brain from potential insults originating from the periphery but is also a bottleneck for drug delivery to the CNS. Our data suggested that injection of NMDA increased delivery of molecules across the BBB, as evidenced by increased presence of IgG. This implies that this may offer a potential mechanisms to increase deliverability of neurotherapeutics, including antibody-based ones.

In conclusion, we have shown that endothelial-NMDA receptor signaling opens the BBB through transcellular pathways that are mediated by molecular signaling pathways directing endosomal trafficking pathways. This endothelial NMDA pathway is not only an important contributor to maintenance of neuronal health but also to vascular integrity. This study is an important initial step in the understanding of the basic mechanism underlying neurovascular coupling and may lead to identification of alternative methods for drug delivery to the CNS.

## Materials and methods

### Reagents and antibodies

N-methyl-D-Aspartate (NMDA), NMDA receptor inhibitor, MK801 (Tocris), PKC inhibitor, Staurosporine (Selleckchem), CaMKII inhibitor, KN-93 (Merck-Millipore) were purchased from manufacturers. Fluo-4 AM, AF594-conjugated Transferrin (Thermoscientific), FITC-ChromPure Mouse Transferrin (Jackson Immunoresearch), and IRDye 800CW 2-DG Optical Probe (Li-Cor) were purchased from manufacturers. Anti-GFAP (Cat# 12389S), NeuN (Cat# 24307S), Caveolin-1 (Cat# 3238) antibodies, Rab5 (Cat# 3547S) were purchased from Cell Signaling Technology. Anti-CD31 antibody (Cat# 550,274, BD Bioscience), Anti-Glucose transporter 1 (GLUT1, Cat# MA1-37,783, Invitrogen), Clathrin Heavy Chain antibodies (Cat# MA1-065, Invitrogen), Anti CD13 (Cat# ab7417, Abcam), and LAMP1 (Cat# sc20011, Santa Cruz Biotechnology) were purchased from respective manufaturer. Dilutions for all antibodies were 1:500. Human primary brain endothelial cells were purchased from Cell Systems (Cat # ACBRI 376). Cells were isolated from microvessels from brains of normal, healthy donor and are positive for CD31, vWF. Experiments were done cells with passage number less than 8 for the maintenance of their properties.

### Animal protocol

NMDA receptor subtype 1 floxed mouse generated by Dr. Susumu Tonegawa [40] were purchased from The Jackson Laboratory (Stock No. 004479) and maintained on C57BL/6 and C3H mixed background which was backcrossed to the C57BL/6 upto 10 generation. Mice were crossed with Tie2-Cre mouse containing the Cre driver specific to the vasculature to generate a mouse specifically lacking NMDA receptor in the brain vasculature. A maximum 5 adult mice in one cage were maintained on a 12 light/12 dark cycle in the specific pathogen free facility. This study was carried out in strict accordance with the recommendations in the Guide for the Care and Use of Laboratory Animals of the National Institutes of Health. The protocol was approved by the Institutional Animal Care and Use Committee of Korea Brain Research Institute (IACUC-17–00,002, IACUC-17–00,012). All animals were immediately sacrificed by cervical dislocation or CO2 inhalation to minimize suffering.

### Designing of conditional knock out mouse of NR1

Transgenic mouse containing the floxed exon sequence of NMDA receptor subtype 1 were purchased from Jackson laboratory (reposited by Dr. Susumu Tonegawa) and were crossed with Tie2-Cre mouse to create conditionally knocked out mouse specific to the endothelial cells. Since NR1 is the core portion of the heteroheteamer of NMDA receptor complexes, knockout of the NR1 is equivalent to NMDA receptor complex knockout. Mice without floxed region from the same litter mate were considered to be the control whereas mice with floxed region with Cre positive genes were considered to be the positive mice. Mice were back crossed more than 10 generations to be used for experiments.

### Live image analysis

For the calcium uptake imaging assay, primary brain endothelial cells were cultured until it reached 100% confluency. Cells were pretreated with Fluo4, a calcium indicator, and incubated for 15 min at 37 °C. Cells were further were treated with 25 uM of NMDA and the level of Ca^2+^ uptake was measured using a Leica TCS SP8 Confocal microscope up to 15 min. The acquired intensity of Fluo4 at each time points was calculated by LAS X software and the values were depicted as a graph using GraphPad 8.0 program.

### Inhibitor assay

To dissect the mechanisms of NMDA receptor mediated regulation of endosomal trafficking pathways, specific inhibitors of CaMKII (KN-93, 2.5 μM for 15 min), protein kinase C (PKC) (Staurosporine, 2 nM for 30 min), and Rho associated kinase (Y27362, 0.5 μM for 30 min) were used to downmodulate each signaling pathway. Subsequently, to analyze endosomal trafficking pathways, cells were treated with NMDA (25 μM) and fixed with 4% PFA in PBS. Cells were further immunostained with anti-LAMP1 and anti-Rab5 (1:500, respectively) antibodies.

### Immunofluorescence assay (IFA)

For immunofluorescence assay on cells, primary brain endothelial cells (Cell Systems) were cultured until they reached 100% confluency. Brain endothelial cells were treated with 25 μM of NMDA or 1 μM of MK801 along with its control at different time points and fixed with 4% PFA for 10 min. Cells were blocked with 5% goat serum and incubated with primary antibodies for 1 h and washed with PBS 3 times 5 min each. Further, cells were incubated with 2ndary antibody conjugated with AF488, 568, and 647 that is specific to the host of primary antibodies. Cells were further washed with PBS 3 times 5 min each and coverslipped with prolong gold. For immunofluorescence assay on the brain section, brains fixed with 4% PFA overnight at 4 °C were sectioned with vibratome with the thickness of 100 μm. Sections were blocked with 5% normal goat serum for 1 h at room temperature and incubated with primary antibodies diluted in 1% Triton X 100/ 1% BSA/ PBS for overnight at room temperature. Subsequently, sections were washed with 0.2% Triton X 100/PBS three times 15 min each. Sections were incubated with secondary antibodies with fluophores in 1% Triton X 100/ 1% BSA/ PBS for overnight at room temperature. Sections were washed with 0.2% Triton X 100/PBS three times 15 min each and was cover-slipped with fluormount G with DAPI (Cat #17,984–24, Electronmicroscope). Images were acquired by Leica SP8 confocal microscope with Z-Stack imaging. Images were reconstructed with maximal projection intensity options using LAS X image software.

### Rho-GTPase activity assay

Human primary brain endothelial cells were treated with 25 μM of of NMDA receptor agonist for different time points and were collected with lysis buffer. Lysates were spun down at 12,000 g for 10 min and were incubated with Rho-Binding domain for overnight at 4’C. Then beads were washed with wash buffer for two times by spinning them at 5,000 g for 2 min. Beads were loaded onto 10% SDS PAGE gel and were ran for 2 h at 100 V and samples were transferred to the nitrocellulose paper for 45 min at 70 V. Blots were incubated with anti-Rho A antibody for 4 h at room temperature and was washed with TBST 1 once for 1 min and incubated with 2ndary mouse antibody conjugated with HRP. Blot was finally washed with TBST for 3 times 5 min each and was visualized with chemiluminescent solution using chemi-doc. Intensity of active RhoA was normalized with that of total RhoA to measure the activity of the RhoA.

### In vivo BBB permeability assay

To test the delivery of glucose to the brain by treatment of NMDA, mice were simultaneously injected with near infrared 2-deoxyglucose (NIR-2DG) with or without NMDA (0.5 mg/kg). Briefly, we have injected 10 nmol of NIR-2DG intravenously, with or without NMDA. The brain was collected and washed three times with ice-cold PBS. The frozen brain was sectioned at the thickness of 10 μm with cryostat microtome and transferred to the glass slide and scanned with Odyssey IR scanner (LI-COR Biotechnology, NE, USA). Intensity of NIR-2DG was quantified with Image J. For transferrin transmigration assay, mice were treated with 10 μg of FITC-Transferrin with NMDA (0.5 mg/kg) or NMDA along with MK801 (0.5 mg/kg) for 1 min. Mice were euthanized and brains were washed 3 times with ice cold PBS and were fixed with 4% PFA. Brain samples were sectioned with vibratome at the thickness of 50 μm and visualized with Leica SP8 confocal microscope (Leica Microsystems Inc, IL, USA). For the assay of the IgG transcytosis, mice were treated with NMDA (0.5 mg/kg), MK801 (0.5 mg/kg), and NMDA along with MK801 (0.5 mg/kg each) for 1 min and were euthanized and brain was collected and fixed with 4% PFA. Further, brain was sectioned with vibratome at the thickness of 50 μm and blocked with 5% normal goat serum (NGS) for 30 min and stained with AF488 conjugated anti-mouse IgG for overnight at room temperature (1:500 in 1% BSA/1% Triton X/PBS) and washed with 0.2% Triton X for 3 times (30 min each). Sections were coverslipped with Fluormount G and visualized with Leica SP8 confocal microscope. Images were reconstructed with maximal projection intensity options using LAS X image software.

### Alcian blue staining

For vessel staining, 4% PFA fixed brain samples were rinsed with 3% acetic acid for three times and the samples were stained with 1% Alcian blue solution (pH 2.5, Cat # B8438, Sigma) for 30 min at room temperature. Sections were further washed briefly with 3% acetic acid and subsequently washed in the running tap water. Stained samples were rinsed with distilled water and dehydrated with 95% and absolute alcohols. Finally, samples were coverslipped with fluormount-G (Cat #17,984–25, Electronmicroscope).

### In vitro BBB permeability assay

To test the effect of the NMDA receptor signaling on the permeability of the brain endothelial cells, we have cultured primary brain endothelial cells on the transwell insert with porous membranes (0.4 μm, Corning, Cat # 353,493) pre-coated with attachment factors (4Z0-201, Cell Systems). Cells were treated with the NMDA (25 μM) or NMDA and MK801 (25 μM and 1 μM, respectively) along with 10 μg/ml of AF594 conjugated transferrin (Cat # T13343, Thermofisher) for upto 15 min. At different time points, media at the bottom chamber was collected. Fluorescence intensity of the AF594 conjugated transferrin that crossed the membrane were measured with excitation at 590 nm and emission at 617 nm with a Flex-Station 3 (Molecular Devices, CA, USA).

### Western blotting

20 ug of samples were loaded onto 10% SDS PAGE ran for 1.5 h at 100 V and subsequently transferred to the nitrocellulose paper for 2 h at 100 V. Nitrocellulose paper was blocked with 1% BSA and incubated with primary antibodies for overnight at 4 °C. Samples were washed with 0.1% TBST for 5 min 3 times each and incubated with HRP conjugated 2ndary antibody for 1 h at room temperature and washed with 0.1% TBST for 5 min 3 times and images were obtained using Fusion chemidoc (Vilber, France).

### Statitstical analysis

Statistical significance was determined by two tailed unpaired t-test comparing two different groups and p values less than 0.05 were considered to be statistically significant. For survival curve, Log-rank (Matel-Cox) test analysis was used and p values less than 0.05 were considered to be statistically significant. All analysis was performed with GraphPad 8.0 program.

## Supplementary Information

**Additional file 1: Figure S1.** Characterization of the primary brain endothelial cell used in the study.Expression of tight junction protein, zona occludensa (ZO1), adhesion molecule (CD31), accumulation of acetylated low density lipoprotein(Ac-LDL), and expression of von Willebrand Factor (vWF) showing the characteristics of the brain endothelial cells were confirmed. Scale bar indicates 75μm. **Figure S2.** Effect of NMDA receptor activation on the integrity of the brain endothelial cell Monolayer.Primary brain endothelial cell monolayer were activated with 25μM NMDA for 1minute and stained with ZO-1 (Green) to visualize tight junction.Scale ba rindicates 50 μm. **Figure S3.** Time dependent effect of NMDA receptor activation on the permeability to FITC-Dextran. HBMVEC cells cultured on the porous membrane were activated with different concentration of NMDA (5,10, and 25 μM) for upto 15 min and concentration of FITC-Dextran at the bottom chambers were quantified and depicted as a graph (n=4). **Figure S4.** Time dependent changes of the BBB permeability to IgG upon NMDA activation. Activation of the NMDA receptor induces increased permeability to the immunoglobulin G from peripheral system in a time dependent manner. C57/BL6 mouse was injected retro-orbitally with PBS, NMDA for 1,5, and 15 min. Brain sections were stained with IgG (Red) to visualize immunoglobulin G. Scale bar indicates 200 μm. **Figure S5.** Activation of NMDA receptor induces permeability changes of the BBB in a heterogenous manner. Activation of the NMDA receptor induces increased permeability to the immunoglobulin G from peripheral system.C57/BL6 mouse was injected retro-orbitally with PBS, NMDA for 1 min. Brain sections were stained with CD31 (Green) and IgG (Red) to visualize endothelial cells and immunoglobulin G, respectively. Images were taken from Cortex, Hippocampus, and Ventral Striatum region. Scale bar indicates 200 and 75 μm at the top and bottom panel. **Figure S6.** Activation of NMDA receptor and its effect on markers for the BBB at the capillary level in the mouse hippocampus. (A–D) C57/BL6 mouse was injected retro-orbitally with PBS(A), NMDA (0.5 mg/kg) (B), NMDA with MK801 (0.5 mg/kgeach) (C), and MK801 (0.5 mg/kg) (D) for 1 min and mice were sacrificed and fixed with 4% PFA overnight. Sections were stained with different markers for the blood brain barrier (BBB), CD31 (Green,1:500), CD13 (Gray,1:500), and GFAP (Red,1:500), a marker for endothelial cells, pericyte, and astrocyte, respectively. Magnified images of each treatment are displayed at the bottom panel. Scale bar indicates 200 μm and 75 μm for upper and bottom panel, respectively. **Figure S7.** Activation of NMDA receptor and its effect on markers for the BBB at the capillary level in the mouse ventral striatum. (A–D) C57/BL6 mouse was injected retro-orbitally with PBS(A), NMDA (0.5 mg/kg) (B ), NMDA with MK801 (0.5 mg/kgeach) (C), and MK801 (0.5 mg/kg) (D) for 1 min and mice were sacrificed and fixed with 4% PFA overnight. Sections were stained with different markers for the blood brain barrier (BBB), CD31 (Green,1:500), CD13 (Gray,1:500), and GFAP (Red,1:500), a marker for endothelial cells, pericyte, and astrocyte, respectively. Magnified images of each treatment are displayed at the bottom panel. Scale bar indicates 200 μm and 75 μm for upper and bottom panel, respectively. Activation of NMDA receptor and absence of its toxic effect on neuronal cells. C57/BL6 mouse was injected retro-orbitally with PBS (A), NMDA (0.5 mg/kg) for 1 min and mice were sacrificed and fixed with 4% PFA overnight. Sections were stained with NeuN (yellow), a marker for neuronal cells. Scale bar indicates 500μm. **Figure S8.** Activation of NMDA receptor and absence of its inflammatory responses in the brain. C57/BL6 mouse was injected retro-orbitally with PBS or NMDA (0.5 mg/kg) for 1 min. (A) Fresh frozen brain sections were stained with anti-CD45 (Green) and anti-CD31 (Red), marker of peripheral immune cells and brain endothelial cells, respectively. Scale bar indicates 75 μm. (B) Fresh frozen sections were stained with anti-intercellular adhesion molecule 1 (ICAM1, red) to test the increased level of adhesion molecule. Scale bar indicates 75 μm. Activation of NMDA receptor reduces lectin and glycocalyx in the brain endothelial cells. C57/BL6 mouse was injected retro-orbitally with PBS or NMDA (0.5 mg/kg) for 1 min. (A) PFA fixed brain sections were stained with AF488-conjugated wheat germ agglutinin (Green) to stain lectin. Scale bar indicates 75 μm. (B) PFA fixed brain section was stained with alcian blue to visualize glycocalyx. Arrow indicates the presence of brain vasculature stained with alcian blue. Scale bar indicates 75 μm.

## Data Availability

Data will be provided by corresponding authors upon proper request.

## Abbreviations

BBB: Blood brain barrier
NMDA: N-methyl-D-aspartate
PKC: Protein Kinase C
NR1: NMDA receptor subunit 1
CaMKII: Ca2 + /calmodulin-dependent protein kinase II
