# Supplementary material for: Activation of NMDA receptors in brain endothelial cells increases transcellular permeability
Source: Fluids Barriers CNS. 2022 Sep 6;19:70. doi: 10.1186/s12987-022-00364-6 (PMC9450318; doi:10.1186/s12987-022-00364-6)
Supplement: Supplementary file 1 — Additional file 1: Figure S1. Characterization of the primary brain endothelial cell used in the study.Expression of tight junction protein, zona occludensa (ZO1), adhesion molecule (CD31), accumulation of acetylated low density lipoprotein(Ac-LDL), and expression of von Willebrand Factor (vWF) showing the characteristics of the brain endothelial cells were confirmed. Scale bar indicates 75μm. Figure S2. Effect of NMDA receptor activation on the integrity of the brain endothelial cell Monolayer.Primary brain endothelial cell monolayer were activated with 25μM NMDA for 1minute and stained with ZO-1 (Green) to visualize tight junction.Scale ba rindicates 50 μm. Figure S3. Time dependent effect of NMDA receptor activation on the permeability to FITC-Dextran. HBMVEC cells cultured on the porous membrane were activated with different concentration of NMDA (5,10, and 25 μM) for upto 15 min and concentration of FITC-Dextran at the bottom chambers were quantified and depicted as a graph (n=4). Figure S4. Time dependent changes of the BBB permeability to IgG upon NMDA activation. Activation of the NMDA receptor induces increased permeability to the immunoglobulin G from peripheral system in a time dependent manner. C57/BL6 mouse was injected retro-orbitally with PBS, NMDA for 1,5, and 15 min. Brain sections were stained with IgG (Red) to visualize immunoglobulin G. Scale bar indicates 200 μm. Figure S5. Activation of NMDA receptor induces permeability changes of the BBB in a heterogenous manner. Activation of the NMDA receptor induces increased permeability to the immunoglobulin G from peripheral system.C57/BL6 mouse was injected retro-orbitally with PBS, NMDA for 1 min. Brain sections were stained with CD31 (Green) and IgG (Red) to visualize endothelial cells and immunoglobulin G, respectively. Images were taken from Cortex, Hippocampus, and Ventral Striatum region. Scale bar indicates 200 and 75 μm at the top and bottom panel. Figure S6. Activation of NMDA receptor and its [file 12987_2022_364_MOESM1_ESM.pdf]

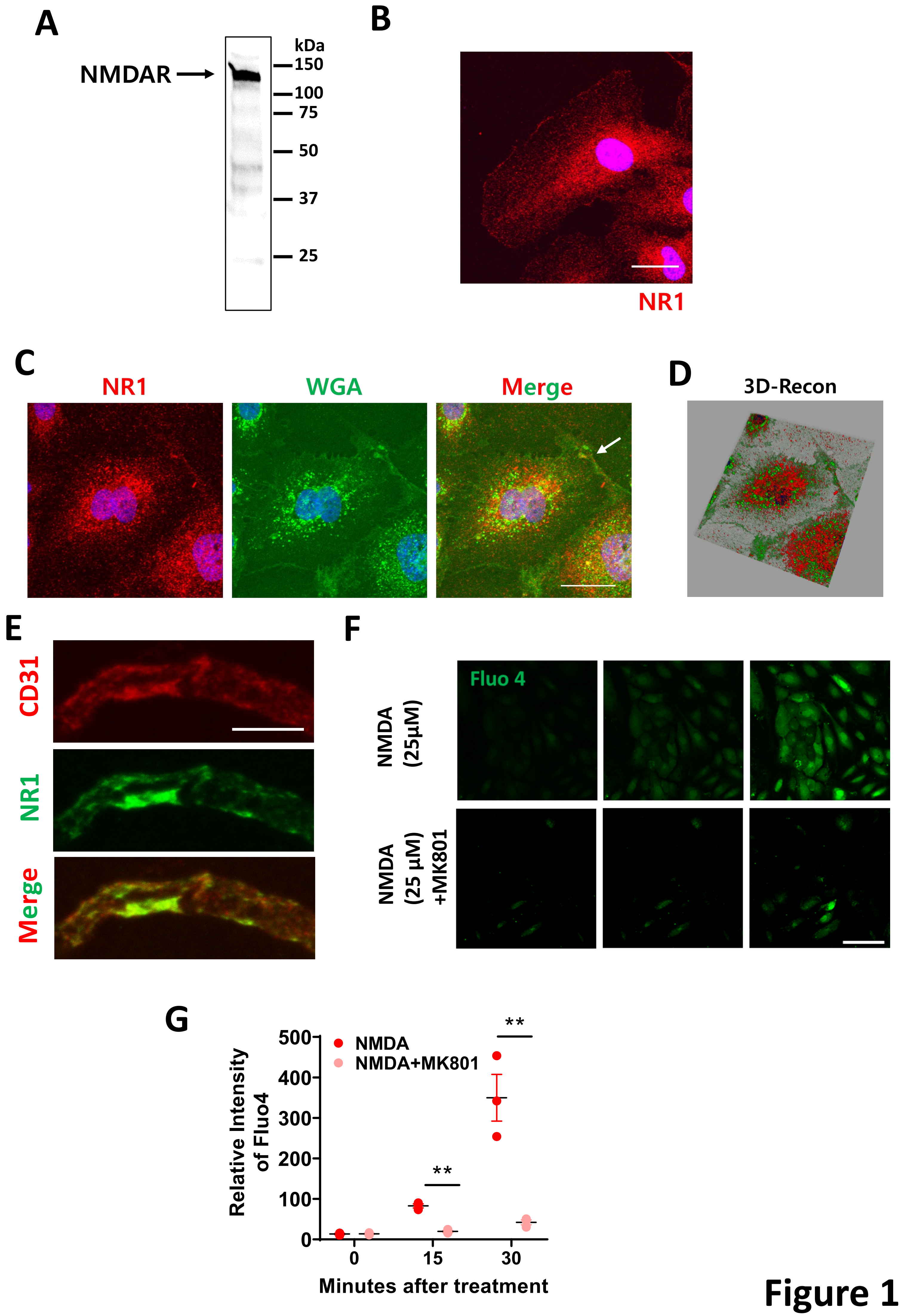
A
NMDAR
kDa
150
100
75
50
37
25
B
NR1
C
NR1
WGA
Merge
D
3D-Recon
E
CD31
NR1
Merge
F
Fluo 4
NMDA (25μM)
NMDA (25 μM) +MK801
G
NMDA
NMDA+MK801
Relative Intensity of Fluo4
Minutes after treatment
**
**
0
15
30
500
400
300
200
100
0

Figure 1

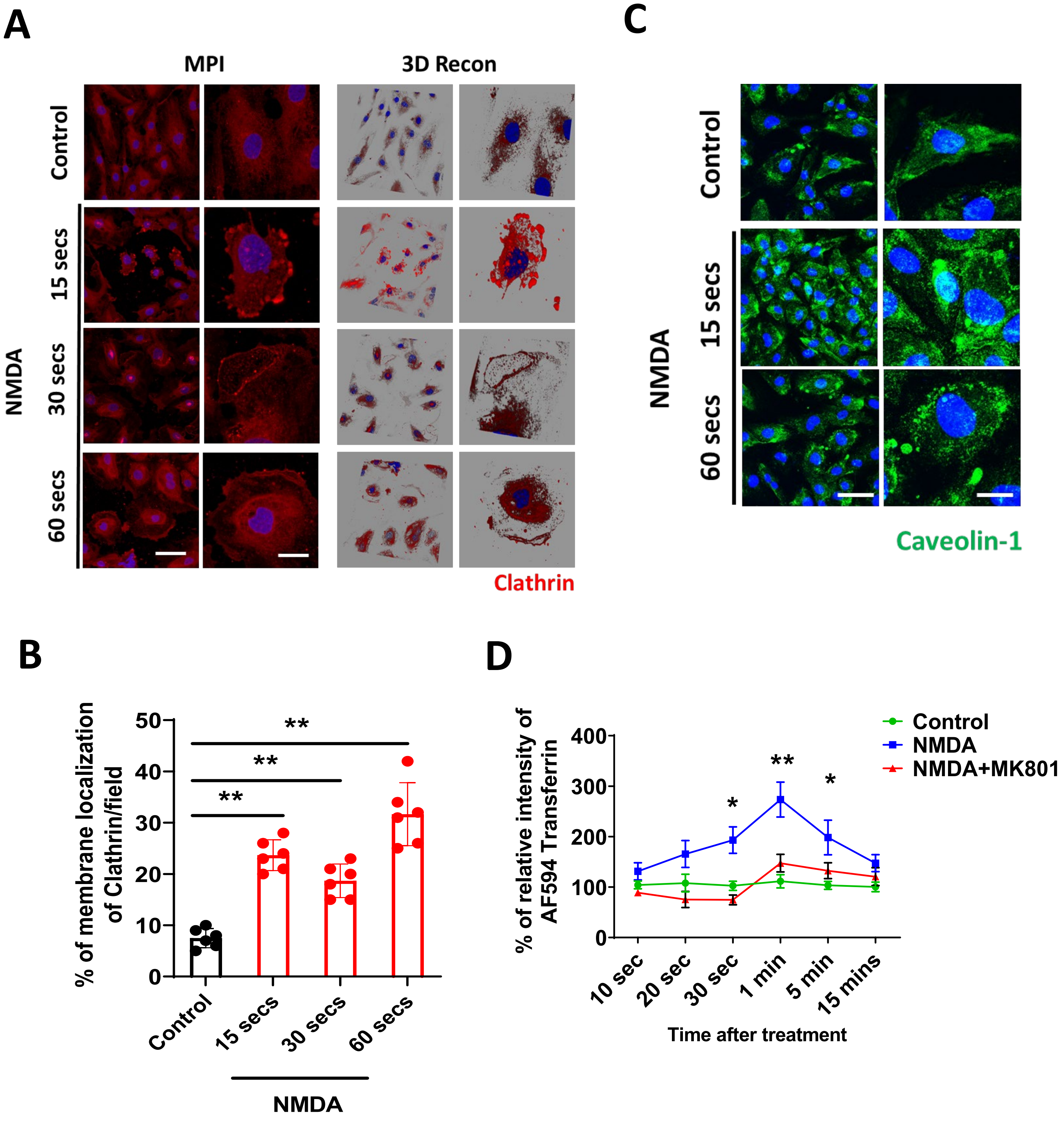
A
MPI
3D Recon
Control
NMDA
15 secs
30 secs
60 secs
Clathrin
C
Control
NMDA
15 secs
60 secs
Caveolin-1
B
% of membrane localization of Clathrin/field
50
40
30
20
10
0
**
**
**
Control
15 secs
30 secs
60 secs
NMDA
D
% of relative intensity of AF594 Transferrin
400
300
200
100
0
Control
NMDA
NMDA+MK801
*
**
*
10 sec
20 sec
30 sec
1 min
5 min
15 mins
Time after treatment

Figure 2

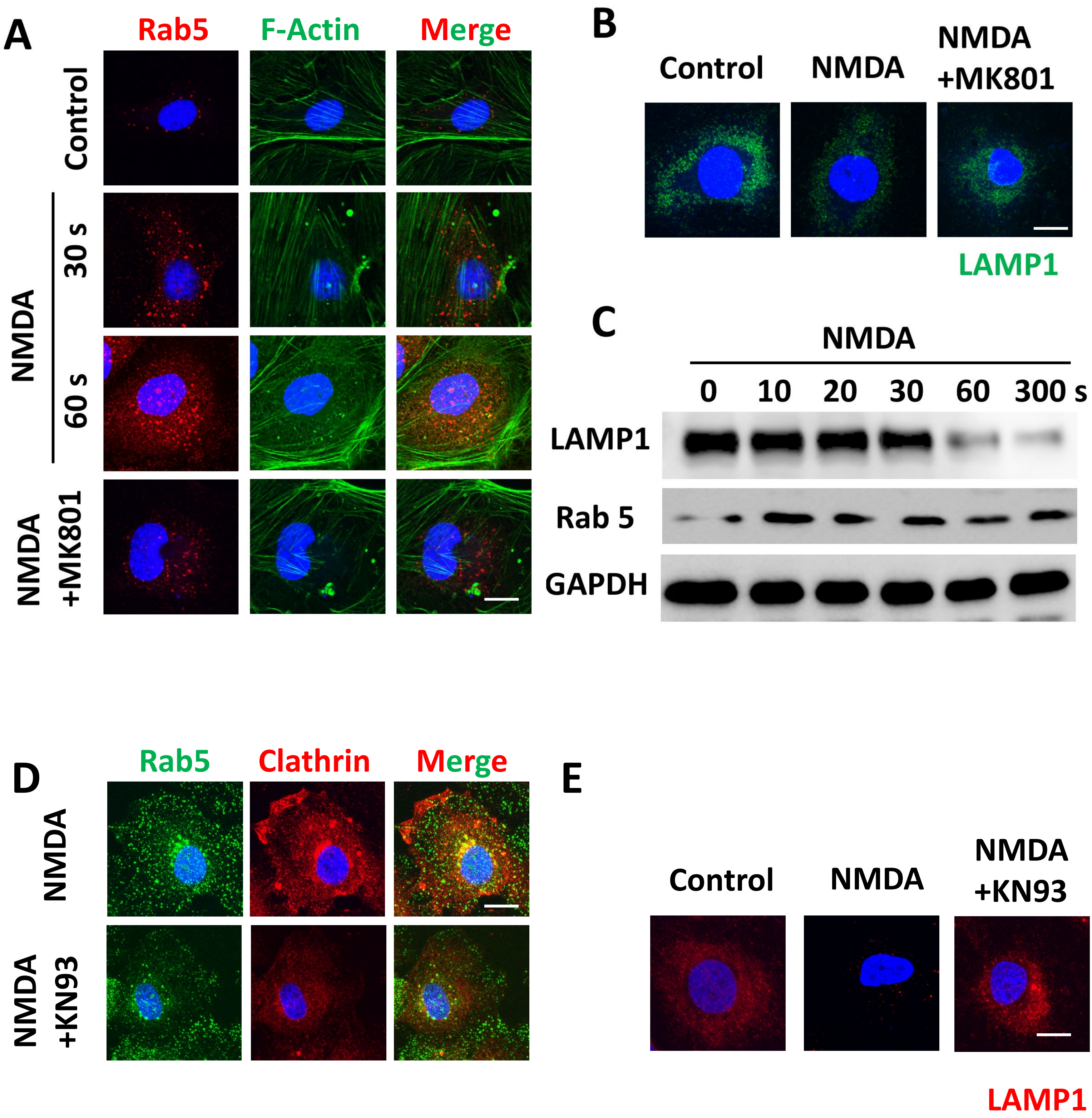
A
Rab5
F-Actin
Merge
Control
NMDA
30 s
60 s
NMDA
+MK801
B
Control
NMDA
NMDA
+MK801
LAMP1
C
NMDA
0
10
20
30
60
300 s
LAMP1
Rab 5
GAPDH
D
Rab5
Clathrin
Merge
NMDA
NMDA
+KN93
E
Control
NMDA
NMDA
+KN93
LAMP1

Figure 3

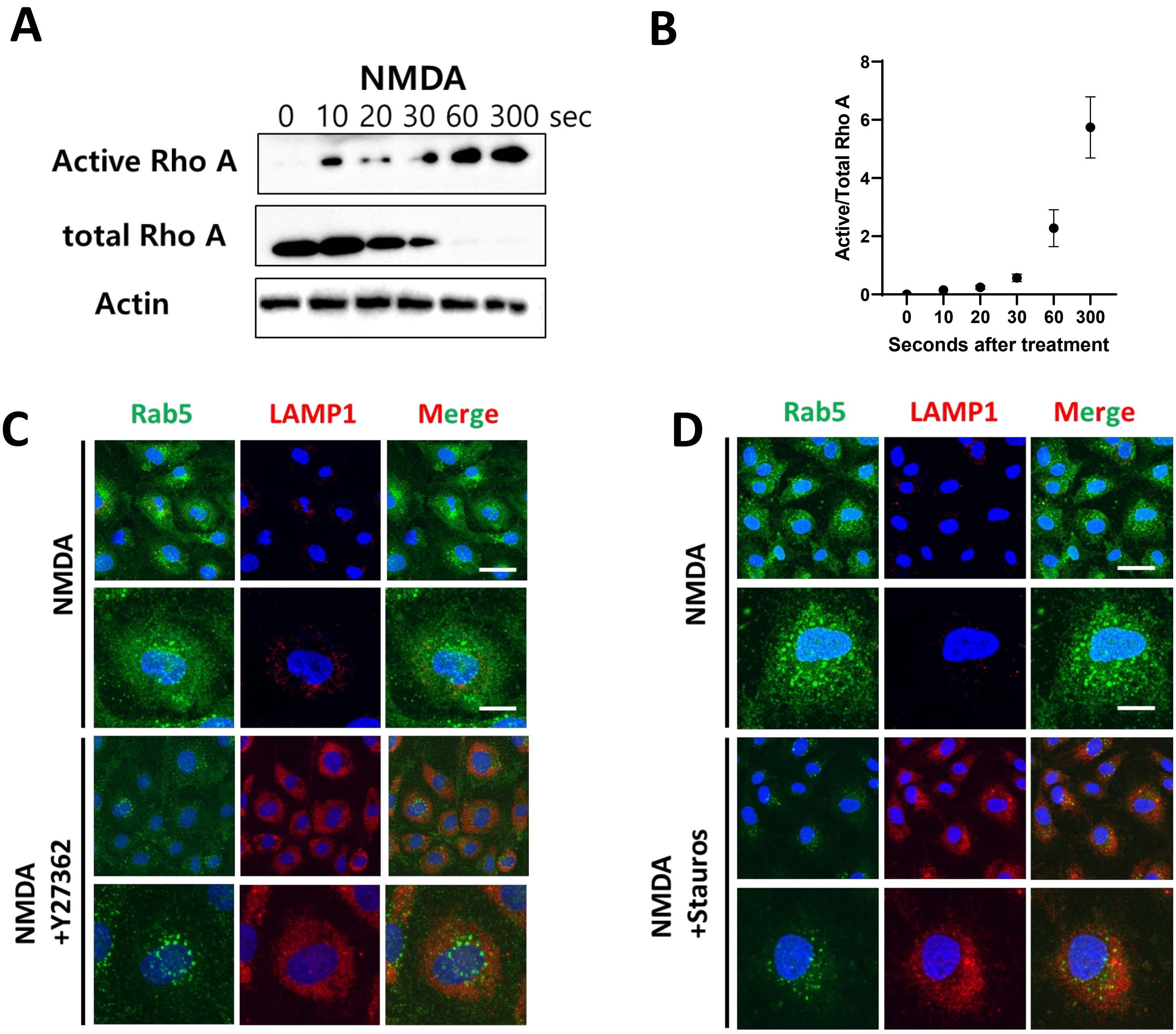
A
NMDA
0 10 20 30 60 300 sec
Active Rho A
total Rho A
Actin
B
Active/Total Rho A
0
2
4
6
8
0 10 20 30 60 300
Seconds after treatment
C
Rab5
LAMP1
Merge
NMDA
NMDA +Y27362
D
Rab5
LAMP1
Merge
NMDA
NMDA +Stauros

Figure 4

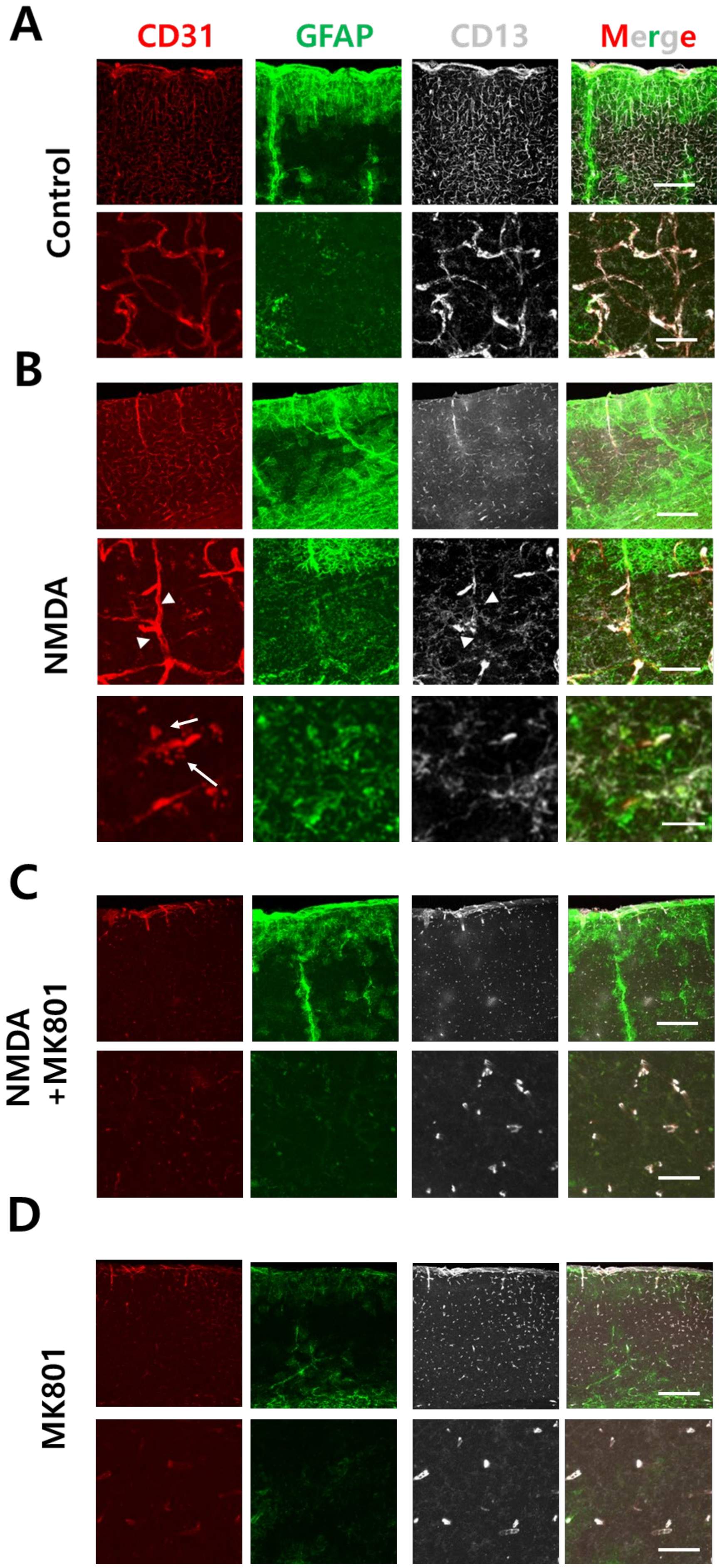

Figure 5

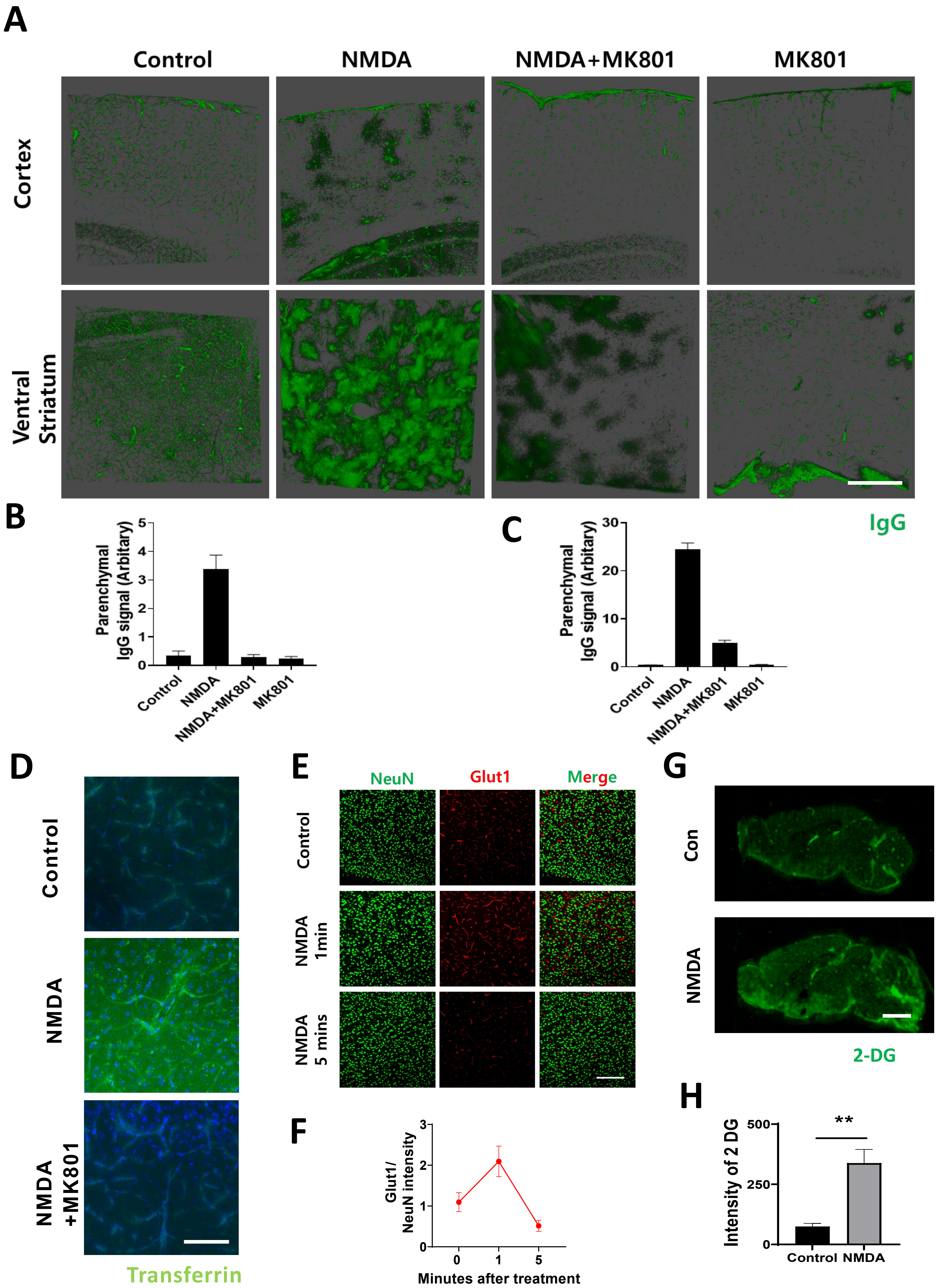
A
Control
NMDA
NMDA+MK801
MK801
Cortex
Ventral Striatum
B
Parenchymal IgG signal (Arbitary)
Control
NMDA
NMDA+MK801
MK801
C
Parenchymal IgG signal (Arbitary)
IgG
D
Control
NMDA
NMDA +MK801
Transferrin
E
NeuN
Glut1
Merge
Control
NMDA 1min
NMDA 5 mins
F
Glut1/ NeuN intensity
Minutes after treatment
G
Con
NMDA
2-DG
H
Intensity of 2 DG
**
Control
NMDA

Figure 6

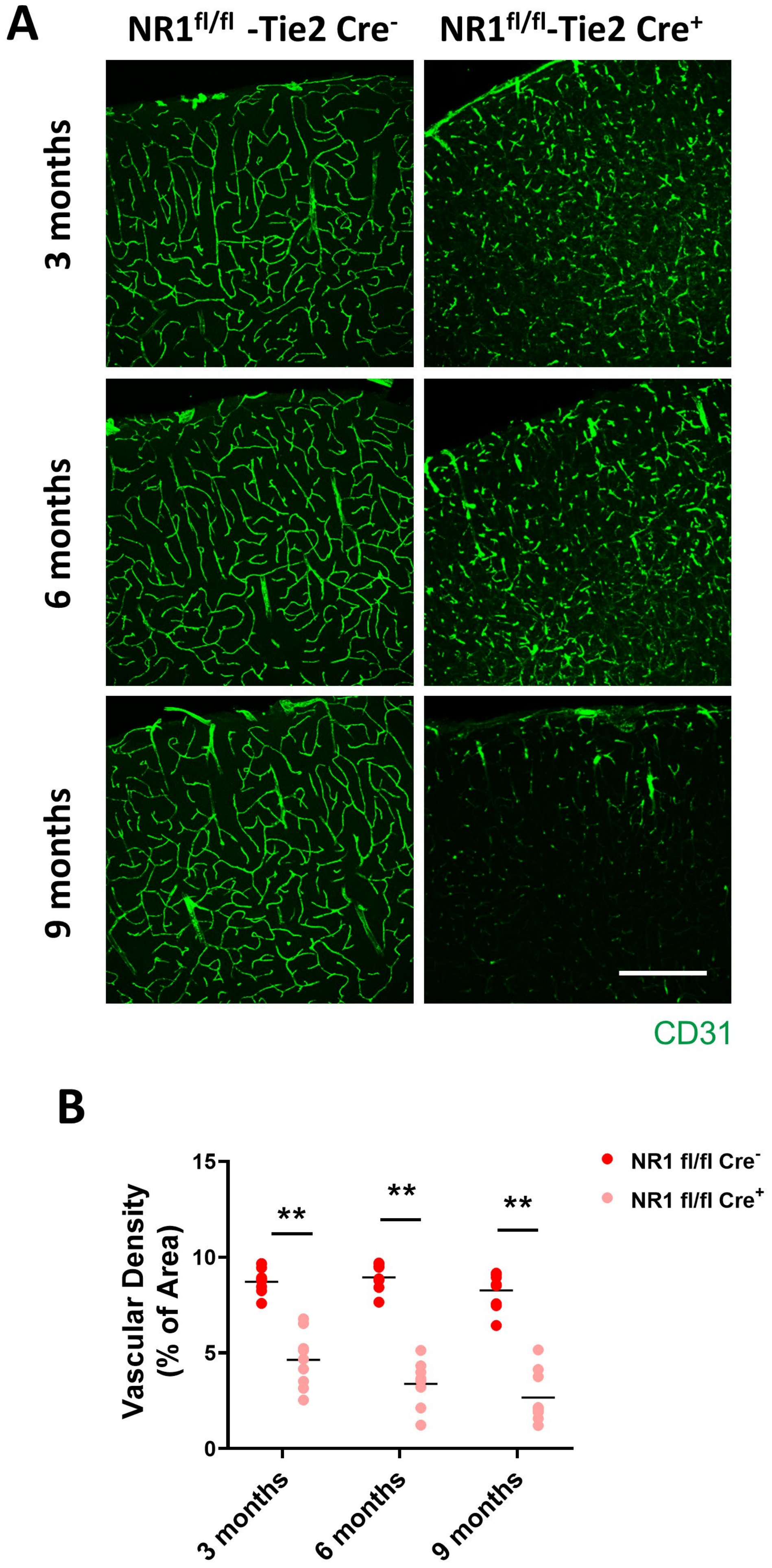

A
NR1fl/fl -Tie2 Cre-
NR1fl/fl-Tie2 Cre+
3 months
6 months
9 months
CD31
B
Vascular Density (% of Area)
15
10
5
0
**
**
**
3 months
6 months
9 months
NR1 fl/fl Cre-
NR1 fl/fl Cre+

Figure 7

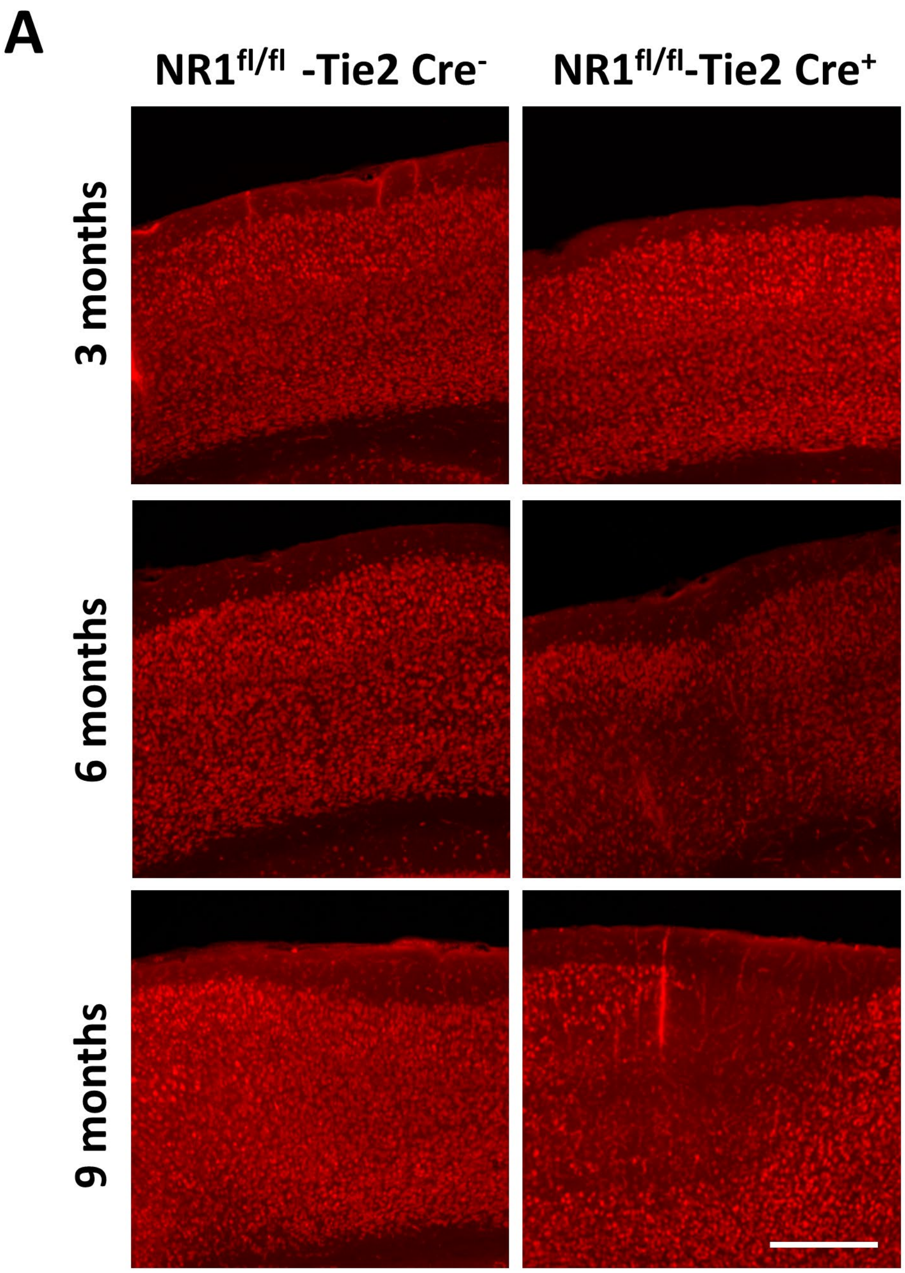
A
NR1fl/fl -Tie2 Cre-
NR1fl/fl-Tie2 Cre+
3 months
6 months
9 months

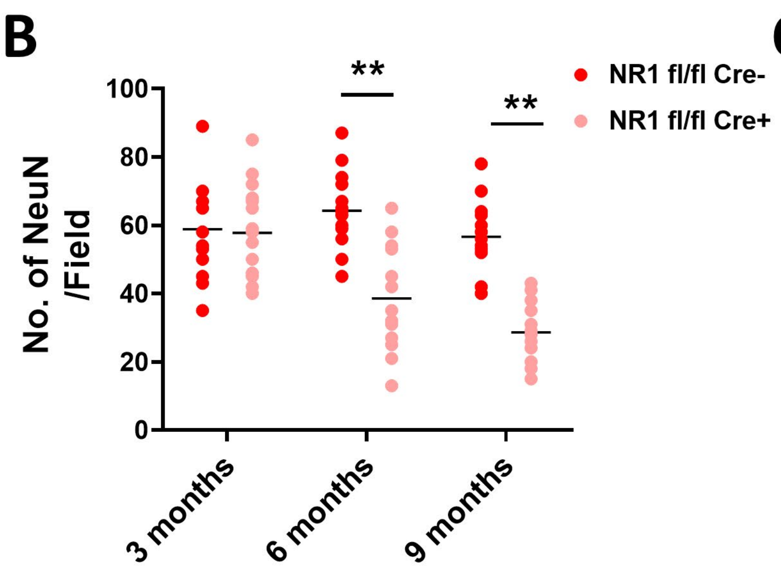
B
No. of NeuN /Field
NR1 fl/fl Cre-
NR1 fl/fl Cre+
**
**
3 months
6 months
9 months

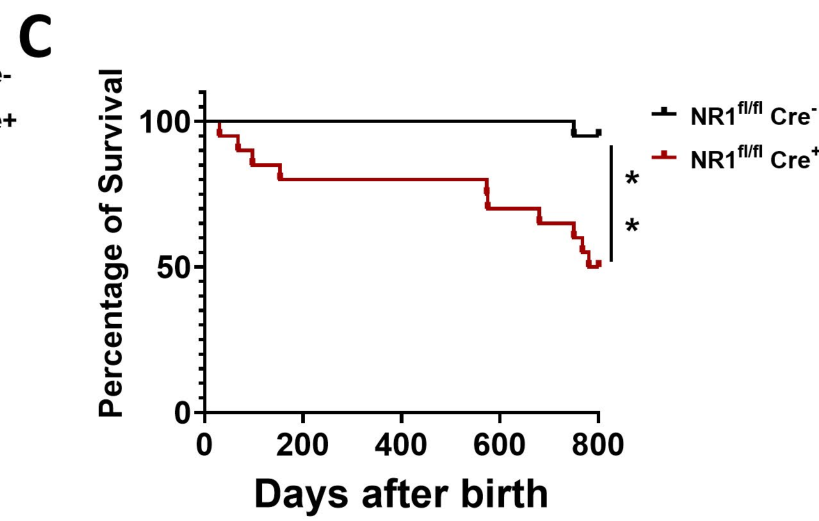
C
Percentage of Survival
Days after birth
NR1fl/fl Cre-
NR1fl/fl Cre+
*
*

Figure 8

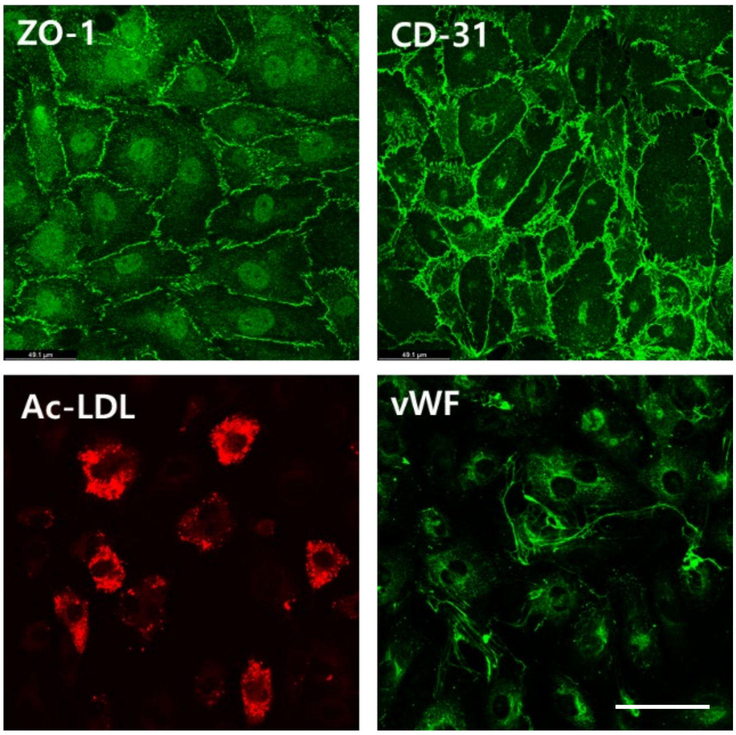

Supplementary Figure 1. Characterization of the primary brain endothelial cell used in the study. Expression of tight junction protein, zona occludensa (ZO1), adhesion molecule (CD31), accumulation of acetylated low density lipoprotein(Ac-LDL), and expression of von Willebrand Factor (vWF) showing the characteristics of the brain endothelial cells were confirmed. Scale bar indicates 75 μm

Supplementary figure 1

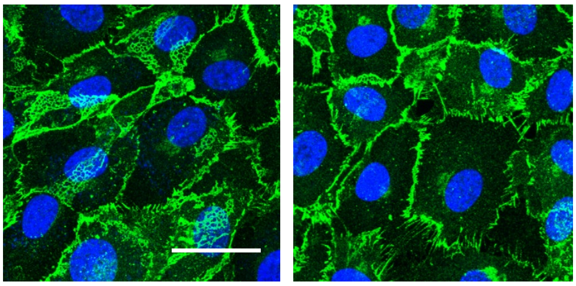

Supplementary Figure 2. Effect of NMDA receptor activation on the integrity of the brain endothelial cell Monolayer. Primary brain endothelial cell monolayer were activated with 25 µM NMDA for 1 minute and stained with ZO-1 (Green) to visualize tight junction. Scale bar indicates 50 µm.

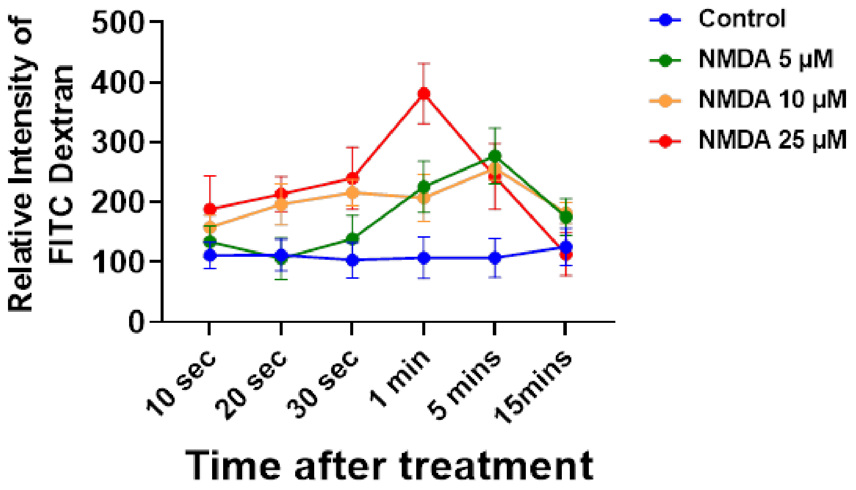

Supplementary Figure 3. Time dependent effect of NMDA receptor activation on the permeability to FITC-Dextran. HBMVEC cells cultured on the porous membrane were activated with different concentration of NMDA (5, 10, and 25 μM) for upto 15 minutes and concentration of FITC-Dextran at the bottom chambers were quantified and depicted as a graph (n=4).

Supplementary figure 3

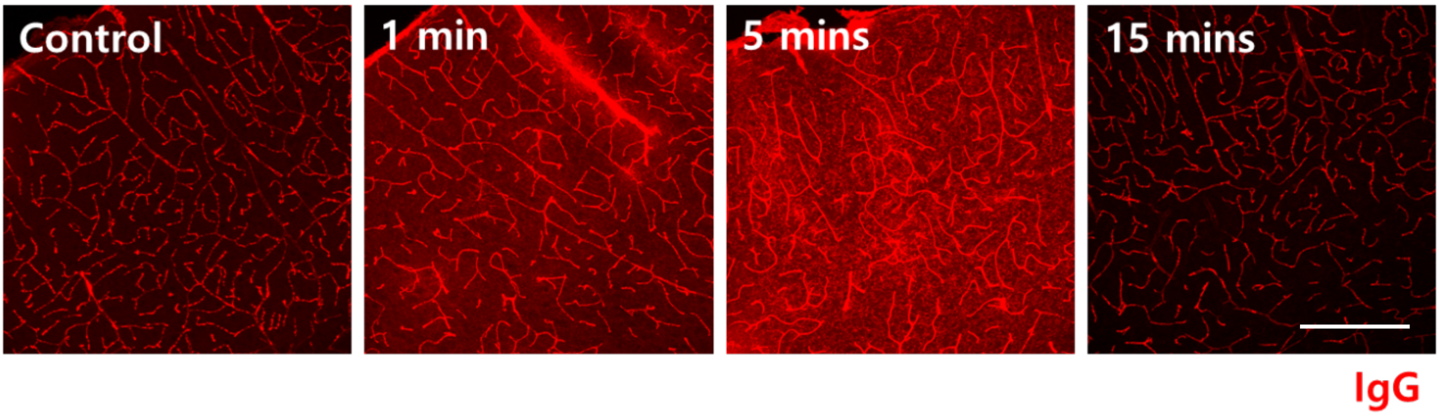

Supplementary Figure 4. Time dependent changes of the BBB permeability to IgG upon NMDA activation. Activation of the NMDA receptor induces increased permeability to the immunoglobulin G from peripheral system in a time dependent manner. C57/BL6 mouse was injected retro-orbitally with PBS, NMDA for 1,5, and 15 minutes. Brain sections were stained with IgG (Red) to visualize immunoglobulin G. Scale bar indicates 200 µm.

Supplementary figure 4

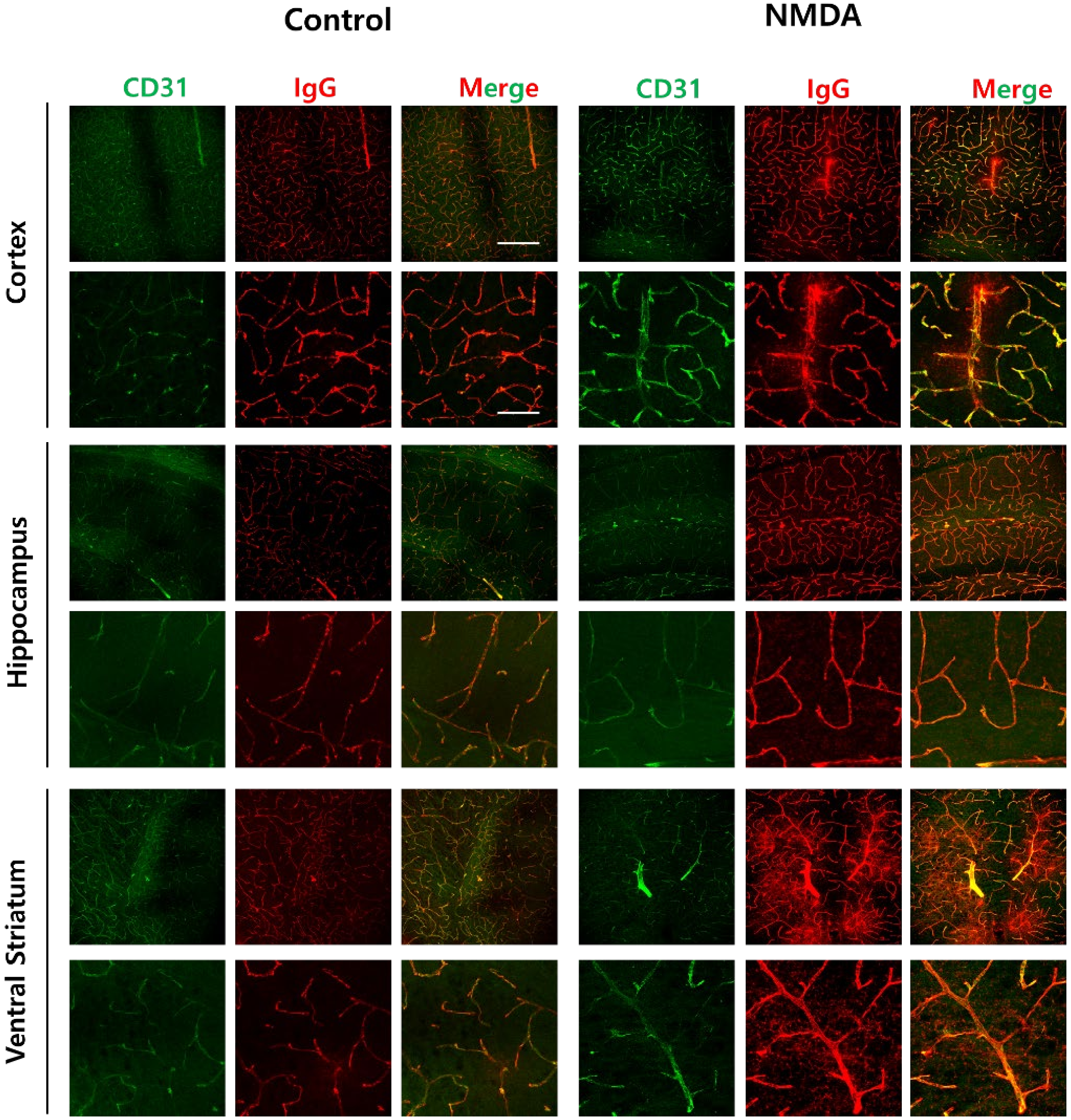

Supplementary Figure 5. Activation of NMDA receptor induces permeability changes of the BBB in a heterogenous manner. Activation of the NMDA receptor induces increased permeability to the immunoglobulin G from peripheral system. C57/BL6 mouse was injected retro-orbitally with PBS, NMDA for 1 minute. Brain sections were stained with CD31 (Green) and IgG (Red) to visualize endothelial cells and immunoglobulin G, respectively. Images were taken from Cortex, Hippocampus, and Ventral Striatum region. Scale bar indicates 200 and 75 µm at the top and bottom panel.

Supplementary figure 5

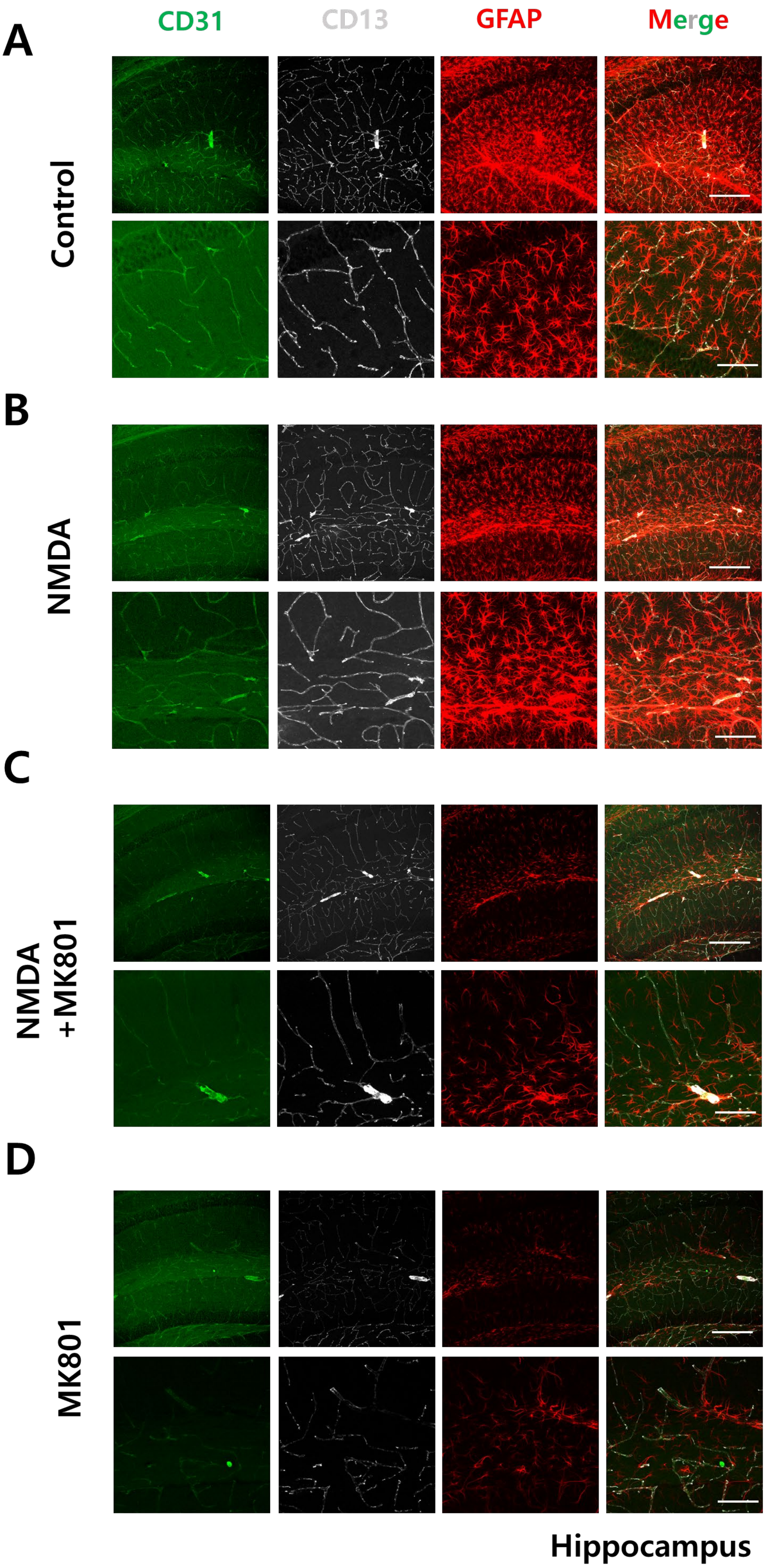

Supplementary Figure 6. Activation of NMDA receptor and its effect on markers for the BBB at the capillary level in the mouse hippocampus. (A-D) C57/BL6 mouse was injected retro-orbitally with PBS (A), NMDA (0.5 mg/kg) (B), NMDA with MK801 (0.5 mg/kg each) (C), and MK801 (0.5 mg/kg) (D) for 1 minute and mice were sacrificed and fixed with 4 % PFA overnight. Sections were stained with different markers for the blood brain barrier (BBB), CD31 (Green, 1:500), CD13 (Gray, 1:500), and GFAP (Red,1:500), a marker for endothelial cells, pericyte, and astrocyte, respectively. Magnified images of each treatment are displayed at the bottom panel. Scale bar indicates 200 μm and 75 μm for upper and bottom panel, respectively.

Supplementary figure 6

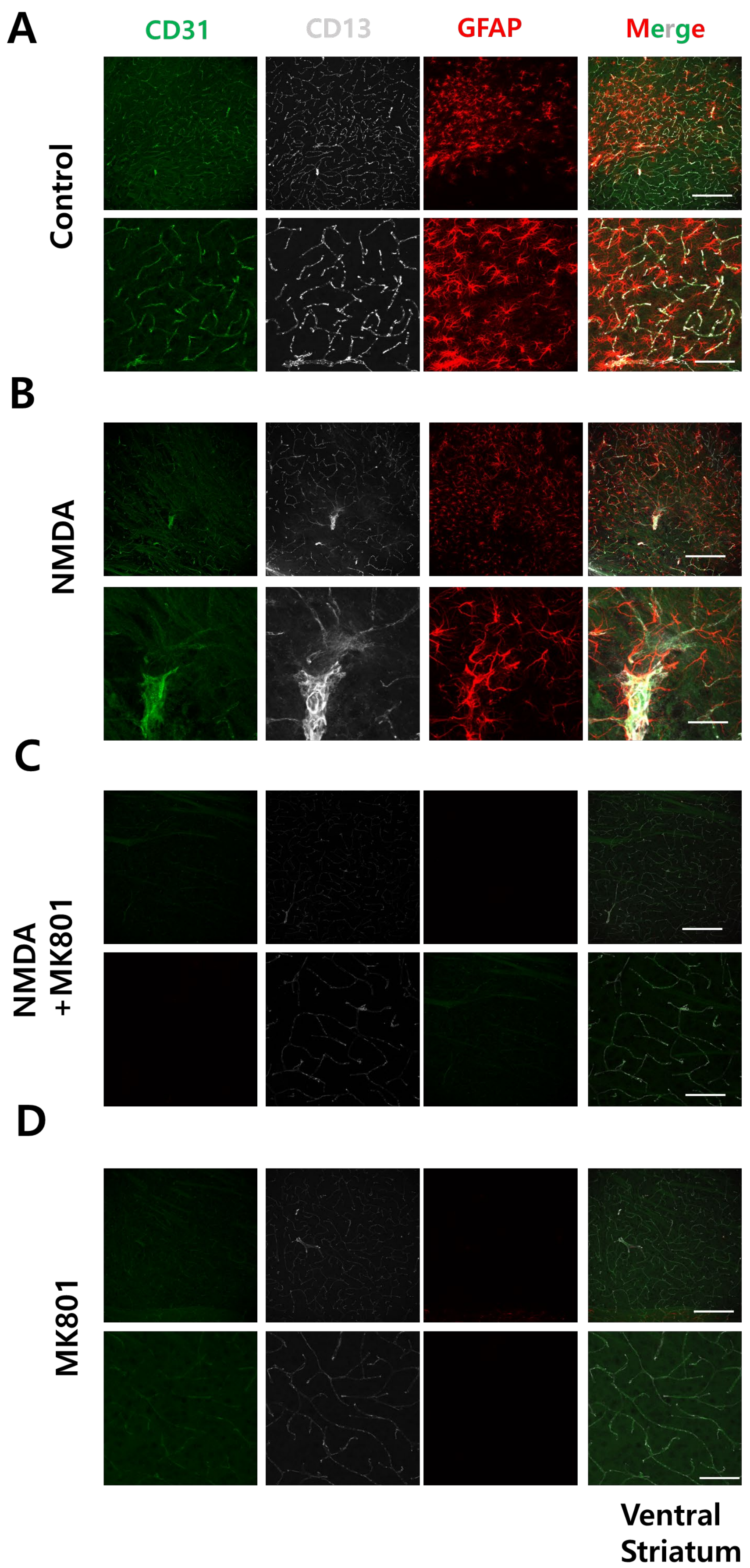

Supplementary Figure 7. Activation of NMDA receptor and its effect on markers for the BBB at the capillary level in the mouse ventral striatum. (A-D) C57/BL6 mouse was injected retro-orbitally with PBS (A), NMDA (0.5 mg/kg) (B), NMDA with MK801 (0.5 mg/kg each) (C), and MK801 (0.5 mg/kg) (D) for 1 minute and mice were sacrificed and fixed with 4 % PFA overnight. Sections were stained with different markers for the blood brain barrier (BBB), CD31 (Green, 1:500), CD13 (Gray, 1:500), and GFAP (Red,1:500), a marker for endothelial cells, pericyte, and astrocyte, respectively. Magnified images of each treatment are displayed at the bottom panel. Scale bar indicates 200 µm and 75 µm for upper and bottom panel, respectively.

Supplementary figure 7

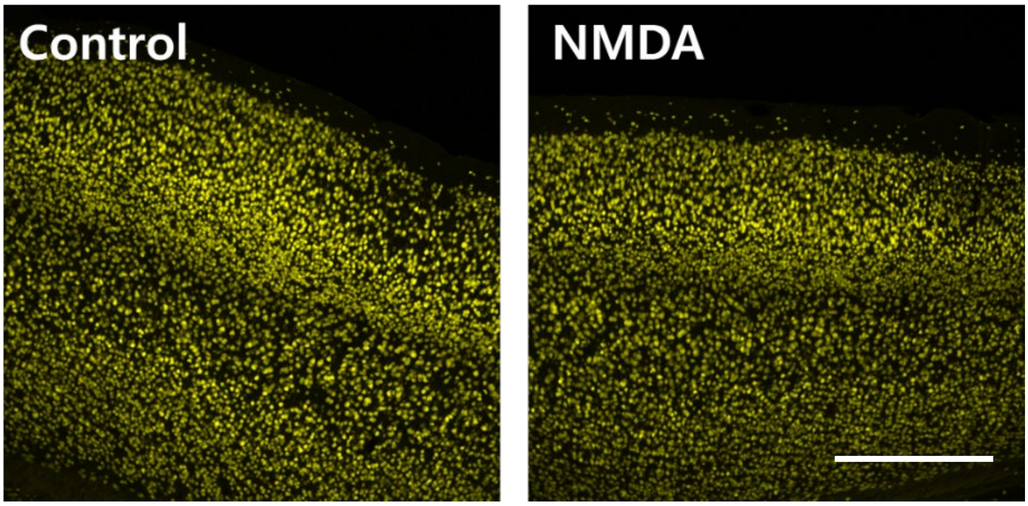

Supplementary Figure 7. Activation of NMDA receptor and absence of its toxic effect on neuronal cells. C57/BL6 mouse was injected retro-orbitally with PBS (A), NMDA (0.5 mg/kg) for 1 minute and mice were sacrificed and fixed with 4 % PFA overnight. Sections were stained with NeuN (yellow), a marker for neuronal cells. Scale bar indicates 500 µm.

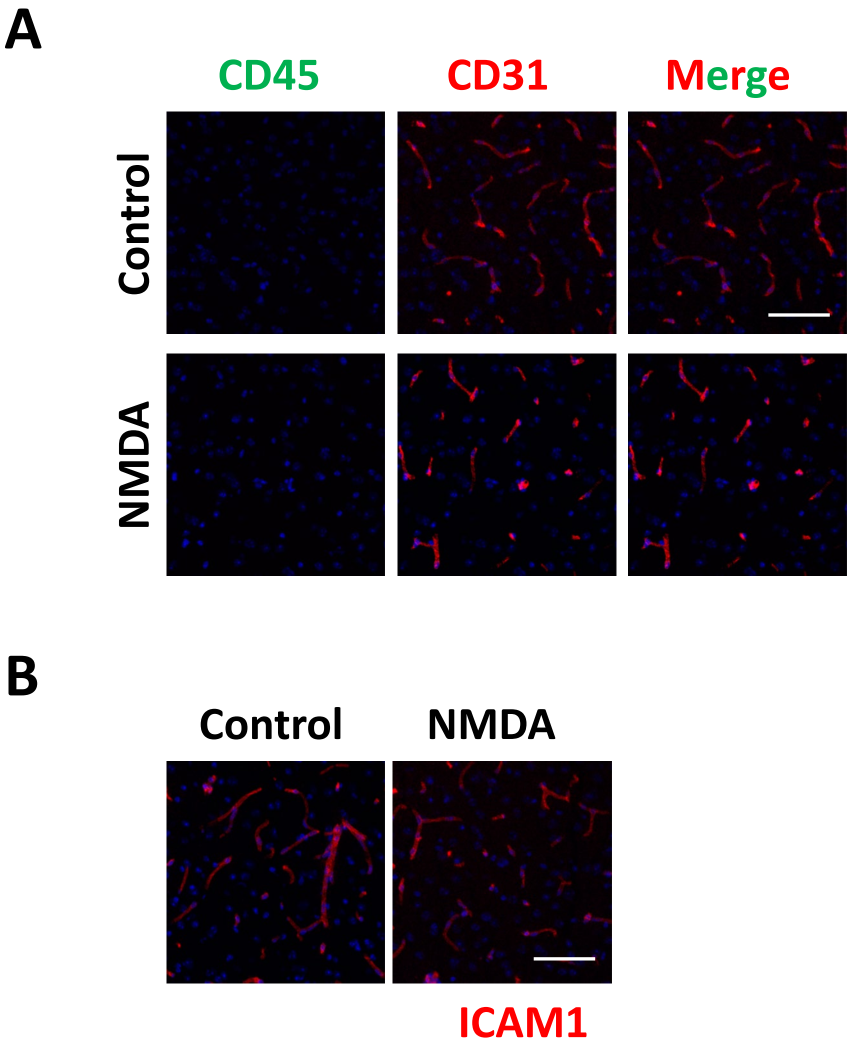

Supplementary Figure 8. Activation of NMDA receptor and absence of its inflammatory responses in the brain. C57/BL6 mouse was injected retro-orbitally with PBS or NMDA (0.5 mg/kg) for 1 minute. (A) Fresh frozen brain sections were stained with anti-CD45 (Green) and anti-CD31 (Red), marker of peripheral immune cells and brain endothelial cells, respectively. Scale bar indicates 75 µm. (B) Fresh frozen sections were stained with anti-intercellular adhesion molecule 1 (ICAM1, red) to test the increased level of adhesion molecule. Scale bar indicates 75 µm.

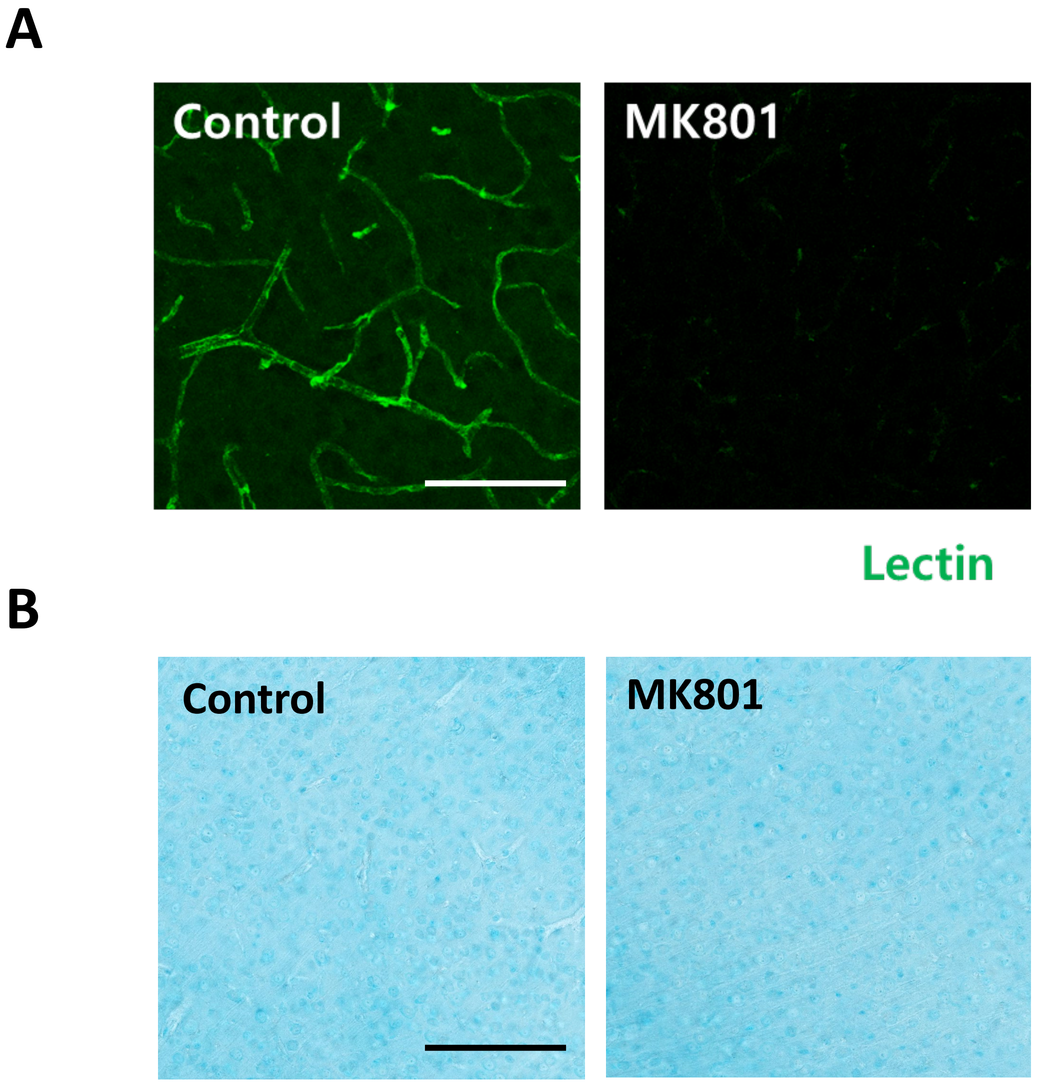

Supplementary Figure 8. Activation of NMDA receptor reduces lectin and glycocalyx in the brain endothelial cells. C57/BL6 mouse was injected retro-orbitally with PBS or NMDA (0.5 mg/kg) for 1 minute. (A) PFA fixed brain sections were stained with AF488-conjugated wheat germ agglutinin (Green) to stain lectin. Scale bar indicates 75 µm. (B) PFA fixed brain section was stained with alcian blue to visualize glycocalyx. Arrow indicates the presence of brain vasculature stained with alcian blue. Scale bar indicates 75 µm.
